# Near-infrared duocarmycin photorelease from a Treg-targeted antibody-drug conjugate improves efficacy of PD-1 blockade in syngeneic murine tumor models

**DOI:** 10.1080/2162402X.2024.2370544

**Published:** 2024-06-20

**Authors:** Hiroshi Fukushima, Aki Furusawa, Seiichiro Takao, Ebaston Thankarajan, Michael P Luciano, Syed Muhammad Usama, Makoto Kano, Shuhei Okuyama, Hiroshi Yamamoto, Motofumi Suzuki, Miyu Kano, Peter L Choyke, Martin J Schnermann, Hisataka Kobayashi

**Affiliations:** a Molecular Imaging Branch, Center for Cancer Research, National Cancer Institute, NIH, Bethesda, MD, USA; b Chemical Biology Laboratory, Center for Cancer Research, National Cancer Institute, NIH, Frederick, MD, USA

**Keywords:** Antibody-drug conjugate, near-infrared light, PD-1 blockade, photouncaging, regulatory T cell

## Abstract

Regulatory T cells (Tregs) play a crucial role in mediating immunosuppression in the tumor microenvironment. Furthermore, Tregs contribute to the lack of efficacy and hyperprogressive disease upon Programmed cell death protein 1 (PD-1) blockade immunotherapy. Thus, Tregs are considered a promising therapeutic target, especially when combined with PD-1 blockade. However, systemic depletion of Tregs causes severe autoimmune adverse events, which poses a serious challenge to Treg-directed therapy. Here, we developed a novel treatment to locally and predominantly damage Tregs by near-infrared duocarmycin photorelease (NIR-DPR). In this technology, we prepared anti-CD25 F(ab’)_2_ conjugates, which site-specifically uncage duocarmycin in CD25-expressing cells upon exposure to NIR light. *In vitro*, CD25-targeted NIR-DPR significantly increased apoptosis of CD25-expressing HT2-A5E cells. When tumors were irradiated with NIR light *in vivo*, intratumoral CD25^+^ Treg populations decreased and Ki-67 and Interleukin-10 expression was suppressed, indicating impaired functioning of intratumoral CD25^+^ Tregs. CD25-targeted NIR-DPR suppressed tumor growth and improved survival in syngeneic murine tumor models. Of note, CD25-targeted NIR-DPR synergistically enhanced the efficacy of PD-1 blockade, especially in tumors with higher CD8^+^/Treg PD-1 ratios. Furthermore, the combination therapy induced significant anti-cancer immunity including maturation of dendritic cells, extensive intratumoral infiltration of cytotoxic CD8^+^ T cells, and increased differentiation into CD8^+^ memory T cells. Altogether, CD25-targeted NIR-DPR locally and predominantly targets Tregs in the tumor microenvironment and synergistically improves the efficacy of PD-1 blockade, suggesting that this combination therapy can be a rational anti-cancer combination immunotherapy.

## Introduction

Regulatory T cells (Tregs) play a crucial role in mediating immunosuppression in the tumor microenvironment.^1,2^ They dampen anti-cancer immune responses through multiple mechanisms: limiting the availability of Interleukin (IL)-2 for effector cells, Cytotoxic-T-lymphocyte-associated protein 4 (CTLA-4)-mediated suppression of antigen-presenting cells, adenosine triphosphate degradation by CD39 and CD73, and secreting immunosuppressive cytokines including IL-10 and Transforming growth factor (TGF)-β.^3,4^ Thus, Tregs are considered a promising target in cancer immunotherapy. In clinical practice, anti-CTLA-4 monoclonal antibody, an immune checkpoint inhibitor that not only blocks the CTLA-4 axis but also depletes Tregs, has been utilized as an effective cancer immunotherapy.^5,6^ However, CTLA-4 blockade systemically depletes Tregs and frequently causes serious autoimmune adverse events, resulting in treatment cessation and long-term use of immunosuppressive agents.^7,8^ To overcome these systemic side effects, therapeutic techniques that locally target Tregs are needed.

In addition to anti-CTLA4 monoclonal antibody, another common immune checkpoint inhibitor is anti-Programmed cell death protein 1 (PD-1) monoclonal antibody.^9^ It has shown high rates of objective response and enhanced survival outcomes in patients with solid malignancies.^10,11^ Nevertheless, PD-1 blockade proves ineffectual in at least 40% of patients, among whom some may manifest hyperprogression – a phenomenon characterized by unexpected, rapid disease progression following the initiation of treatment.^12,13^ Recent investigations have revealed that PD-1 expression on CD8^+^ T cells and Tregs, as well as the relative balance of these two cell populations, constitute pivotal factors influencing the effectiveness of PD-1 blockade.^14^ Furthermore, a tumor microenvironment dominated by Tregs can result in hyperprogression subsequent to PD-1 blockade.^15,16^ These data provide a rationale for combining Treg-directed therapy and PD-1 blockade.

Near-infrared (NIR) photocaging groups, which are constructed upon the heptamethine cyanine scaffold, can be delivered to target cells by conjugating them with a targeting moiety such as an antibody.^17^ This facilitates not only fluorescence imaging-based diagnosis but also provides a therapeutic option by site-specifically releasing bioactive compounds upon exposure to NIR light.^17,18^ Because NIR light can penetrate 1 − 2 cm through tissues, NIR photocaging groups have the advantage of clinical applications in humans. We previously developed cyanine photocages, which use a heptamethine cyanine scaffold conjugated with a duocarmycin payload, a derivative of the DNA-alkylating natural product, through a *N,N*’-diethylethylenediamine linker.^19^ This linker is cleaved with 780 nm light, leading to photorelease of duocarmycin. By conjugating with panitumumab, a human Epidermal Growth Factor Receptor (EGFR)-targeted monoclonal antibody, NIR duocarmycin photorelease (NIR-DPR) showed significant anti-cancer efficacy *in vivo*.^19,20^

**Figure 1.**
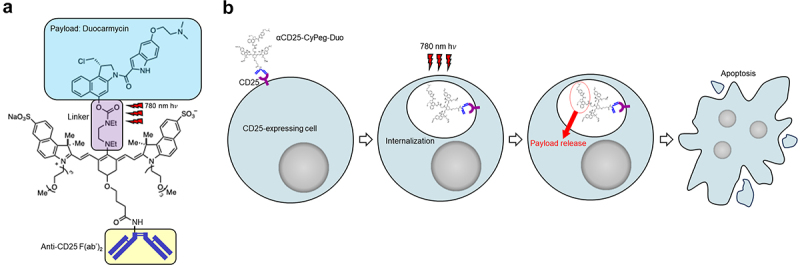
Structure of αCD25-CyPeg-Duo and cytotoxic mechanism of CD25-targeted NIR-DPR. (a) structure of αCD25-CyPeg-Duo. The N,N’-diethylethylenediamine linker is cleaved with 780 nm hv. (b) cytotoxic mechanism of CD25-targeted NIR-DPR. After binding of αCD25-CyPeg-Duo to CD25 on the surface of CD25-expressing cells, it is internalized into the cytoplasm. Light irradiation at 780 nm triggers payload uncaging, which results in apoptosis of CD25-expressing cells.

In this study, we demonstrate a novel therapeutic approach to locally and predominantly damage Tregs by applying NIR-DPR targeted to CD25. We constructed the conjugate of anti-CD25 F(ab’)_2_ with a novel optimized derivative CyPeg-Duo (Figure 1a). CD25, a component of the IL-2 receptor, is one of the optimal targets for Treg-directed therapy because it is highly expressed on intratumoral Tregs.^21,22^ After intravenously administering the αCD25-CyPeg-Duo, the conjugate binds to CD25-expressing cells and are internalized (Figure 1b). Direct exposure to 780 nm light causes photorelease of duocarmycin, resulting in selective apoptosis of CD25-expressing cells (Figure 1b). Using the technology of CD25-targeted NIR-DPR, we successfully achieved a significant decrease in intratumoral CD25^+^ Treg populations resulting in a loss of their immune suppression function. Subsequently, we investigated the therapeutic efficacy of CD25-targeted NIR-DPR in combination with PD-1 blockade using syngeneic murine tumor models.

## Materials and methods

### Synthesis of CyPeg-Duo-conjugated anti-CD25 F(ab’)_2_

Anti-CD25-F(ab′)_2_ was generated from anti-CD25-IgG (clone PC-61.5.3; Bio X Cell, West Lebanon, NH, USA), as described previously.^22^ The CyPeg-Duo-NHS ester (Molecular weight 1773.6921), was synthesized through the route shown in Supplementary Figure S1 based in part on prior work (Supplementary Methods).^17^ CyPeg-Duo-conjugated anti-CD25 F(ab’)_2_ was synthesized as follows.^19,20^ Anti-mouse CD25 F(ab’)_2_ (733 μg, 6.7 nmol) was incubated with ten-fold molar excess of CyPeg-Duo-NHS ester (8 mmol in DMSO) in 100 mM Na_2_HPO_4_ solution (pH 8.5) for one hour at room temperature. The mixture was purified with PD-10 columns containing Sephadex G25 resin (GE Healthcare, Piscataway, NJ, USA). The resulting conjugate is abbreviated as αCD25-CyPeg-Duo (Figure 1a). The concentration of CyPeg-Duo and protein was determined with absorption at 778 nm and 280 nm using UV−vis, respectively (8453 Value System; Agilent Technologies, Santa Clara, CA, USA). The number of CyPeg-Duo molecules per F(ab’)_2_ was calculated from division of those values. An average of two CyPeg-Duo molecules were bound to each anti-CD25-F(ab’)_2_. Success of conjugation was verified by Sodium dodecyl sulfate-polyacrylamide gel electrophoresis (SDS-PAGE; Supplementary Figure S2).

### Cell culture

Murine cell lines, HT2-A5E (T lymphocyte), EL4 (lymphoma), MB49-luc (bladder cancer), MC38 (colon cancer), Pan02-luc (pancreatic cancer), MOC1 (oral cancer), mEERL-hEGFR (hEGFR-expressing oropharyngeal cancer),^23^ MOC2 (oral cancer), and LL/2-luc (lung cancer), were used in this study. HT2-A5E and EL4 were purchased from ATCC (Manassas, VA, USA), MB49-luc and Pan02-luc from GenTarget Inc (San Diego, CA, USA), MOC1 and MOC2 from Kerafast (Boston, MA, USA), and LL/2-luc from Imanis Life Sciences (Rochester, MN, USA). MC38 was kindly provided by Dr. Thomas Waldmann, NIH. mEERL-hEGFR was kindly provided by Dr. Chad Spanos, Sanford Research. EL4, MB49-luc, MC38, Pan02-luc, and LL/2-luc cells were cultured in RPMI1640 medium (Thermo Fisher Scientific, Rockford, IL, USA) supplemented with 10% fetal bovine serum (FBS; Thermo Fisher Scientific) and 100 IU/mL penicillin/streptomycin (Thermo Fisher Scientific). For HT2-A5E cells, 0.05 mmol 2-mercaptoethanol and 0.1 nmol human IL-2 were also added. MOC1 and MOC2 cells were cultured in the mixture of IMDM medium and Ham’s Nutrient Mixture F12 Media (at a ratio of 2:1, GE Healthcare) supplemented with 5% FBS, 100 IU/mL penicillin/streptomycin, 5 ng/mL insulin (MilliporeSigma, Burlington, MA, USA), 40 ng/mL hydrocortisone (MilliporeSigma), and 3.5 ng/mL human recombinant EGF (MilliporeSigma). mEERL-hEGFR cells were grown in DMEM/F-12 (Thermo Fisher Scientific) supplemented with 10% FBS, 100 IU/mL penicillin/streptomycin, and 1% human keratinocyte growth supplement (Thermo Fisher Scientific). All cells were cultured in a humidified incubator at 37°C in an atmosphere of 95% air and 5% carbon dioxide for no more than 30 passages.

### In vitro NIR-DPR

Cells (1 × 10^5^) were incubated in 100 μL of culture medium containing αCD25-CyPeg-Duo (1.9 μg/mL) for six hours at 37°C. After washing with PBS, phenol-red-free medium was added. NIR laser-light (780 nm, 50 J/cm^2^, 150 mW/cm^2^) was applied using an ML6600 laser system (Modulight, Tampere, Finland). Eighteen hours after irradiation, cells were analyzed by apoptosis assay. In the apoptosis assay, cells were stained with Annexin V (STEMCELL Technologies, Seattle, WA, USA) and Fixable Viability Dye (Thermo Fisher Scientific) in Annexin V binding buffer (STEMCELL Technologies). The fluorescence of cells was measured using BD FACSLyric (BD Biosciences, San Jose, CA, USA) and FlowJo software (FlowJo LLC, Ashland, OR, USA). Total apoptotic cells were calculated by summing early (Annexin V^+^/Fixable Viability Dye^−^) and late apoptotic cells (Annexin V^+^/Fixable Viability Dye^+^).

### Animal models

Six- to eight-week-old female immunocompetent C57BL/6 mice (strain #000664) were purchased from the Jackson Laboratory (Bar Harbor, ME, USA). The lower part of the body of the mice was shaved before image analysis. MB49-luc (2 × 10^6^), MC38 (2 × 10^6^), Pan02-luc (2 × 10^6^), MOC1 (2 × 10^6^), mEERL-hEGFR (2 × 10^6^), MOC2 (2 × 10^6^), or LL/2-luc (1 × 10^6^) cells were inoculated into the right dorsum. Six (MB49-luc and MC38) or five (LL/2-luc) days after inoculation, mice with tumors reaching 5 mm in diameter were used for the experiments. Mice with the other tumors (Pan02-luc, MOC1, mEERL-hEGFR, and MOC2) were used for the experiments when they reached 5 mm in diameter. Tumor volumes were evaluated three times per week by a 3-dimensional tumor scanning system (TumorImager2, Biopticon, Princeton, NJ, USA). Random mice grouping was performed based on tumor volumes using TumorManager software (Biopticon). Mice were euthanized with carbon dioxide when tumors reached 500 mm^3^ in volume. Alive mice were censored 60 days after tumor inoculation. Tumor disappearance for four weeks or longer after treatment was defined as complete remission (CR).

### Flow-cytometric analysis of immune cells

To evaluate immune cell expressions in the tumor, tumor-draining lymph node (TDLN), or spleen, each was harvested after mice were euthanized. Single-cell suspensions were prepared as described previously.^15,24^ Cells were stained with the following antibodies: anti-CD3e (clone 145-2C11), anti-CD4 (RM4–5), anti-CD11b (M1/70), anti-CD11c (N418), anti-CD19 (6D5), anti-CD25 (3C7), anti-CD45 (30-F11), anti-CD62L (MEL-14), anti-CD69 (H1.2F3), anti-CD86 (GL-1), anti-F4/80 (BM8), anti-IL-10 (JES5-16E3), anti-Ki-67 (10A8), anti-Ly6C (HK1.4), anti-Ly6G (1A8), anti-I-A/I-E (M5/114.15.2), anti-PD-1 (29F.1A12), and rat IgG2bκ isotype control (RTK4530) were from BioLegend (San Diego, CA, USA); anti-CD4 (RM4–5), anti-CD8α (53–6.7), anti-CD19 (eBio1D3 [1D3]), anti-CD40 (1C10), anti-CD44 (IM7), anti-CD45 (clone 30-F11), anti-CD80 (16-10A1), anti-F4/80 (BM8), Foxp3 (FJK16s), anti-Ly6G (1A8), anti-NK1.1 (PK136), and rat IgG2aκ isotype control (eBR2a) were from Thermo Fisher Scientific. Dead cells were gated out by Fixable Viability Dye (Thermo Fisher Scientific). For IL-10 staining, cells were stimulated with eBioscience™ Cell Stimulation Cocktail plus protein transport inhibitors (Thermo Fisher Scientific) at 37°C for four hours before staining. For intracellular staining of IL-10, Ki-67, and Foxp3, cells were fixed and permeabilized with Foxp3 Transcription Factor Staining Buffer Set (Thermo Fisher Scientific). The stained cells were analyzed using FACSLyric (BD Biosciences) and Flowjo software (FlowJo LLC). Gating strategies were shown in Supplementary Figure S3 and S4. Cell types were determined as follows; T cells: CD45^+^/CD3^+^, CD4^+^ T cells: CD45^+^/CD3^+^/CD4^+^/CD8^−^, Tregs: CD45^+^/CD3^+^/CD4^+^/CD8^−^/Foxp3^+^, Helper T cells (Ths): CD45^+^/CD3^+^/CD4^+^/CD8^−^/Foxp3^−^, CD25^+^ Tregs: CD45^+^/CD3^+^/CD4^+^/CD8^−^/Foxp3^+^/CD25^+^, CD8^+^ T cells: CD45^+^/CD3^+^/CD4^−^/CD8^+^, B cells: CD45^+^/CD19^+^, macrophages: CD45^+^/CD11b^+^/(F4/80)^+^, monocytes: CD11b^+^/(F4/80)^−^/Ly6G^−^/Ly6C^+^, dendritic cells (DCs): CD45^+^/(F4/80)^−^/CD11c^+^/(I-A/I-E)^+^, neutrophils: CD11b^+^/(F4/80)^−^/Ly6G^+^/Ly6C^int^, and natural killer (NK) cells: CD45^+^/CD3^−^/NK1.1^+^. Relative fluorescence intensity (RFI) of PD-1 was calculated as the ratio of the mean fluorescence intensity of anti-PD-1 antibody to that of the isotype control. CD8^+^/Treg PD-1 ratio was calculated as the ratio of PD-1 RFI in CD8^+^ T cells to PD-1 RFI in CD25^+^ Tregs.

### In vivo NIR-DPR

To evaluate the therapeutic efficacy of CD25-targeted NIR-DPR, mice were randomized into three groups as follows: (i) no treatment (Control), (ii) intravenous injection of αCD25-CyPeg-Duo (19 μg) without NIR light irradiation (αCD25-CyPeg-Duo IV), and (iii) intravenous injection of αCD25-CyPeg-Duo (19 μg) followed by NIR light irradiation (NIR-DPR). To evaluate the therapeutic efficacy of CD25-targeted NIR-DPR combined with PD-1 blockade, mice were randomized into four groups as follows: (i) no treatment (Control), (ii) intravenous injection of αCD25-CyPeg-Duo (19 μg) followed by NIR light irradiation (NIR-DPR), (iii) intraperitoneal injection of anti-PD-1 antibody (PD-1 blockade), and (iv) intravenous injection of αCD25-CyPeg-Duo (19 μg) followed by NIR light irradiation and intraperitoneal injection of anti-PD-1 antibody (Combination). αCD25-CyPeg-Duo was administered six (MB49-luc and MC38) or five (LL/2-luc) days after inoculation. NIR light (780 nm, 50 J/cm^2^, 150 mW/cm^2^) was administered to the tumors 24 hours after αCD25-CyPeg-Duo administration. During NIR light exposure, mice were covered by aluminum foil except for a hole to expose only the target tumor, thus shielding most of the mouse from NIR light. Mice were intraperitoneally injected with 200 μg or 100 μg of anti-mouse PD-1 monoclonal antibody (clone RMP1–14; Bio X Cell) at indicated timepoints. 800-nm fluorescence and white light images were obtained before and after NIR light irradiation using a Pearl Imager (LI-COR Bioscience, Lincoln, NE, USA). A ROI was placed on the tumor and mean fluorescence intensity was calculated for each ROI. Luciferase activity of tumors was evaluated by bioluminescence imaging (BLI) analysis. D-luciferin (15 mg/mL in 200 μL PBS for MB49-luc, 3 mg/mL in 200 μL PBS for LL/2-luc; Gold Biotechnology, St. Louis, MO, USA) was injected intraperitoneally, and luciferase activity was analyzed with a PRISM in vivo imaging system (MediLumine, Montreal, Canada) and ImageJ (NIH, Bethesda, MD, USA). ROI was placed to include the entire tumor, and luciferase activity was quantified as an integrated density of each ROI for 60 seconds.

### Multiplex immunohistochemistry (IHC)

After harvesting tumors from mice, formalin-fixed, paraffin-embedded (FFPE) sections were prepared. Multiplex IHC was performed as described previously,^25,26^ using Opal Automation IHC Kit (Akoya Bioscience, Menlo Park, CA, USA) and Bond RXm autostainer (Leica Biosystems, Wetzlar, Germany). The sections were stained with 4,6-diamino-2-phenyl indole (DAPI) and the following antibodies: anti-CD8 (clone EPR20305; Abcam, Cambridge, MA, USA), anti-CD4 (EPR19514; Abcam), anti-CD11b (EPR1344; Abcam), anti-digoxigenin (DIG; 9H27L19; Thermo Fisher Scientific), anti-Foxp3 (1054C; Novus Biologicals, Littleton, CO, USA), anti-Granzyme B (GZMB; rabbit poly; Abcam), and anti-pan-cytokeratin (pan-CK; rabbit poly; Bioss Antibodies). Coverslips were mounted using ProLong Diamond Antifade Mountant (Thermo Fisher Scientific). Stained slides were analyzed with Mantra Quantitative Pathology Workstation (Akoya Biosystems) and inForm Tissue Finder software (Akoya Biosystems). inForm software was trained to detect tissues by defining areas with pan-CK expression and other areas as tumor and stroma, respectively. To calculate CD8^+^ T cell density, cell phenotypes were determined as follows: pan-CK^−^/CD8^+^ = CD8^+^ T cells, pan-CK^−^/CD4^+^ = CD4^+^ T cells, and pan-CK^+^ = cancer cells, respectively. Five images were taken from each tumor sample, and cell density was calculated by combining areas for each tissue phenotype and cell counts of each cell phenotype. For the calculation of the density of GZMB^+^CD8^+^ T cells, cell phenotyping and GZMB expression analysis were performed separately for the same set of images, then cell segmentation data were consolidated and analyzed using phenoptrReports and phenoptr (Akoya Biosciences).

### Detection of DIG-labeled F(ab’)_2_ by multiplex IHC

Non-reactive control F(ab’)_2_ was generated from polyclonal rat IgG (Bio X Cell). Anti-CD25 F(ab’)_2_ or control F(ab’)^2^ was labeled with DIG (Thermo Fisher Scientific) by incubating 733 µg of F(ab’)_2_ and 50 µg of DIG, similar to the CyPeg-Duo conjugation. The resulting DIG-labeled F(ab’)_2_ is abbreviated as CD25-DIG and control-DIG, respectively. Tumor-bearing mice were injected with CD25-DIG or control-DIG (19 µg) via lateral tail vein. Tumors were harvested 24 hours after injecting DIG-labeled F(ab’)_2_. The distribution of DIG-labeled F(ab’)_2_ was analyzed in FFPE sections by multiplex IHC using anti-DIG antibody. Cell types were determined as follows: pan-CK^−^/CD8^+^ = CD8^+^ T cells, pan-CK^−^/CD4^+^/Foxp3^+^ = Tregs, pan-CK^−^/CD4^+^/Foxp3^−^ = Ths, pan-CK^−^/CD11b^+^ = myeloid cells, and pan-CK^+^ = cancer cells.

### Statistical analysis

Data are shown as mean ± SEM. GraphPad Prism 10 (GraphPad Software, La Jolla, CA, USA) was used for statistical analysis. An unpaired *t*-test was performed to compare continuous data between the two groups. The chi-square test was used to compare categorical data. A one-way ANOVA followed by Tukey’s test was performed to compare continuous data among multiple groups. Tumor volumes and luciferase activity were compared using repeated measures two-way ANOVA followed by Tukey’s test. Survival percent was determined by a Kaplan-Meier method, and the results were compared using the log-rank test with Bonferroni correction. *p* < 0.05 was defined as statistically significant.

## Results

### Cytotoxic effects of CD25-targeted NIR-DPR on CD25-expressing cells in vitro

**Figure 2.**
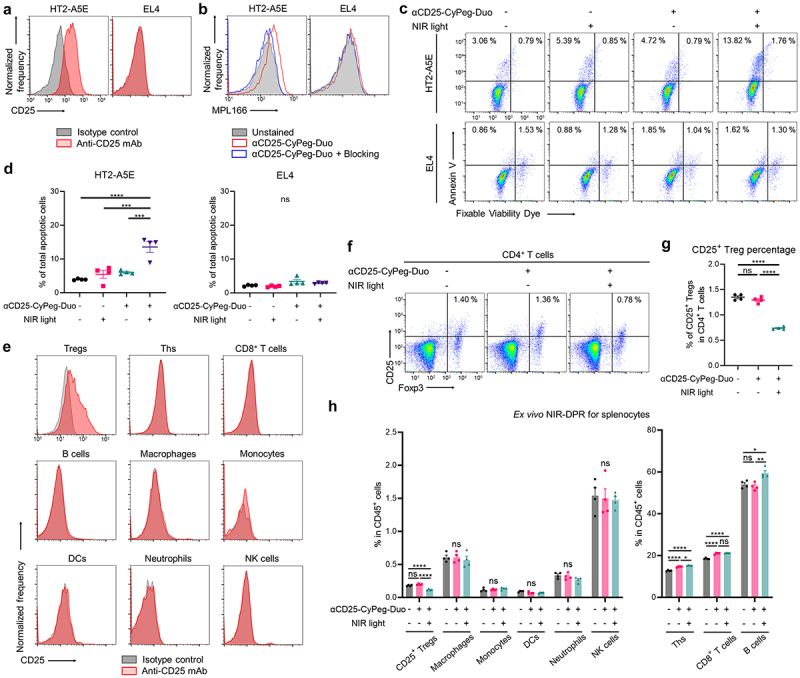
CD25-targeted NIR-DPR showed cytotoxic effects on CD25-expressing cells in vitro and Tregs ex vivo.

Conjugated αCD25-CyPeg-Duo was evaluated by SDS-PAGE. The molecular weight of αCD25-CyPeg-Duo was approximately the same as that of unconjugated anti-CD25 F(ab’)_2_ but 800-nm fluorescence was detected only in αCD25-CyPeg-Duo (Supplementary Figure S2). *In vitro*, we used CD25-expressing HT2-A5E cells and non-CD25-expressing EL4 cells (Figure 2a). In HT2-A5E cells, fluorescence of CyPeg-Duo was detected after incubating with αCD25-CyPeg-Duo, but it was blocked by adding excessive amounts of unconjugated anti-CD25 F(ab’)_2_ (Figure 2b). Meanwhile, no fluorescence of αCD25-CyPeg-Duo was detected in EL4 cells (Figure 2b). This suggests specific binding of αCD25-CyPeg-Duo to CD25-expressing cells.

(a) Flow cytometric analysis of *in vitro* CD25 expression in HT2-A5E or EL4 cells. (b) Detection of αCD25-CyPeg-Duo bound to HT2-A5E or EL4 cells by flow cytometry. (c) and (d), *In vitro* apoptosis assay of HT2-A5E or EL4 cells after CD25-targeted NIR-DPR. Total apoptotic cells were calculated by summing early (Annexin V^+^/Fixable Viability Dye^−^) and late apoptotic cells (Annexin V^+^/Fixable Viability Dye^+^). (c) Representative dot plots. (d) Comparison of the percentage of total apoptotic cells (*n* = 4, mean ± SEM; one-way ANOVA followed by Tukey’s test; ***, *p* < 0.001; ****, *p* < 0.0001; ns, not significant). (e) Flow cytometric analysis of *ex vivo* CD25 expression in splenocytes. Foxp3^+^CD4^+^ T cells highly expressed CD25, whereas other immune cells did not show CD25 expression. (f)–(h), *Ex vivo* CD25-targeted NIR-DPR for splenocytes. (f) Representative dot plots of CD4^+^ T cells. The percentage of CD25^+^ Tregs in CD4^+^ T cells was shown. (g) Comparison of the percentage of CD25^+^ Tregs in CD4^+^ T cells (*n* = 4, mean ± SEM; one-way ANOVA followed by Tukey’s test; ****, *p* < 0.0001; ns, not significant). (h) Comparison of the percentage of each immune cells in CD45^+^ cells (*n* = 4, mean ± SEM; one-way ANOVA followed by Tukey’s test; *, *p* < 0.05; **, *p* < 0.01; ****, *p* < 0.0001; ns, not significant).

Next, cytotoxic efficacy of CD25-targeted NIR-DPR was analyzed in HT2-A5E and EL4 cells *in vitro* (Figure 2c). HT2-A5E cells treated with αCD25-CyPeg-Duo incubation followed by NIR light irradiation showed a significantly higher percentage of total apoptotic cells compared to the control and cells treated with either αCD25-CyPeg-Duo without NIR light irradiation or NIR light irradiation alone (Figure 2d, *left*). Meanwhile, no significant increase in total apoptotic cells was observed after CD25-targeted NIR-DPR in EL4 cells (Figure 2d, *right*).

### CD25-targeted NIR-DPR partially depleted Tregs ex vivo

CD25 expression in splenocytes was evaluated by flow cytometry *ex vivo*. CD25 expression was detected in Tregs, but not in other immune cells (Figure 2e). Thus, cytotoxic effects of CD25-targeted NIR-DPR on CD25^+^ Tregs were analyzed using splenocytes *ex vivo*. The percentage of CD25^+^ Tregs in CD4^+^ T cells was slightly but significantly decreased 24 hours after treatment (Figure 2f,g). Additionally, *ex vivo* CD25-targeted NIR-DPR significantly decreased Ki-67 and IL-10 expressions in CD25^+^ Tregs (Supplementary Figure S5). Meanwhile, CD25-targeted NIR-DPR did not significantly decrease other immune cell populations (Figure 2h).

### Biodistribution and intratumoral delivery of αCD25-CyPeg-Duo

**Figure 3.**
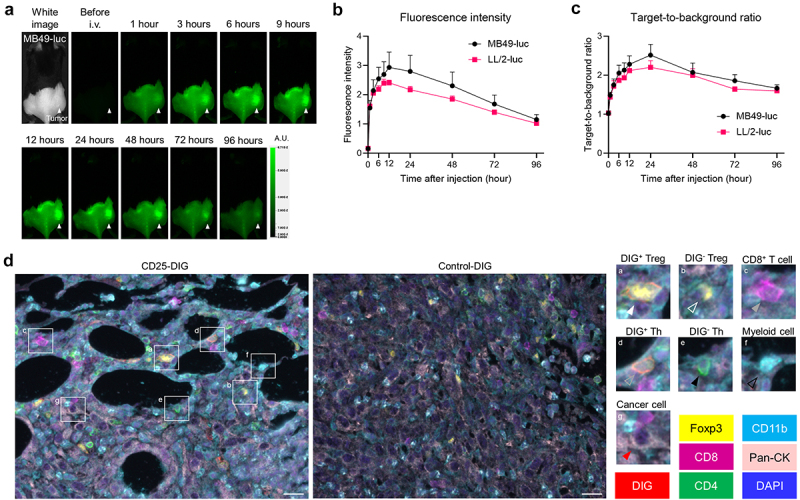
Delivery and biodistribution of intravenously administered αCD25-CyPeg-Duo in vivo.

*In vivo* 800-nm fluorescence of αCD25-CyPeg-Duo was serially analyzed after intravenous injection in MB49-luc and LL/2-luc tumor-bearing mice (Figure 3a). Intratumoral 800-nm fluorescence reached a peak 12 hours after injecting αCD25-CyPeg-Duo, followed by a gradual attenuation over time (Figure 3b). Target-to-background ratio (TBR) of αCD25-CyPeg-Duo increased up to 24 hours after injection and gradually declined thereafter (Figure 3c).

(a)–(c), *In vivo* fluorescence imaging of αCD25-CyPeg-Duo. All images were acquired at the indicated timepoints after intravenously injecting αCD25-CyPeg-Duo into MB49-luc or LL/2-luc tumor-bearing mice. (a) Representative fluorescence images at 800 nm in a MB49-luc tumor-bearing mouse (A.U., arbitrary units). (b) Quantitative analysis of 800-nm fluorescence intensity at the tumor site after intravenously injecting αCD25-CyPeg-Duo (*n* = 7). (c) Quantitative analysis of target-to-background ratio (TBR) after intravenously injecting αCD25-CyPeg-Duo (*n* = 7). (d) *In vivo* delivery of digoxigenin (DIG)-conjugated anti-CD25 F(ab’)_2_ to MB49-luc tumors. Tumors were harvested 24 hours after injecting CD25-DIG or control-DIG into mice and its distribution in MB49-luc tumors was examined by multiplex immunohistochemistry (images; ×200; scale bar, 20 μm). Antibody stainings of DIG, Foxp3, CD8, CD4, CD11b, and pan-cytokeratin (Pan-CK) are shown in red, yellow, magenta, green, cyan, and pink, respectively. Nucleus is stained with DAPI and shown in blue. The insets (a–g) are enlarged and displayed on the right side. They show representative images of a DIG^+^ Treg (a, white-filled arrowhead), DIG^−^ Treg (b, white hollow arrowhead), CD8^+^ T cell (c, gray-filled arrowhead), DIG^+^ Th (d, gray hollow arrowhead), DIG^−^ Th (e, black-filled arrowhead), myeloid cell (f, black hollow arrowhead), and cancer cell (g, red-filled arrowhead).

To evaluate the delivery of anti-CD25 F(ab’)_2_ to Tregs *in vivo*, either CD25-DIG [DIG-labeled anti-CD25 F(ab’)^2^] or control-DIG [DIG-labeled control F(ab’)^2^] was intravenously administered into MB49-luc tumor-bearing mice, then intratumoral DIG distribution was analyzed by multiplex IHC (Figure 3d). CD25-DIG was detected on the cell surface of most Tregs, suggesting the successful delivery of CD25-DIG to CD25^+^ Tregs. CD25-DIG was also detected on the cell surface of a subset of Ths, suggesting the presence of CD25^+^ Ths in the tumor. CD25-DIG was not detected on any CD8^+^ T cells, myeloid cells, or cancer cells. Therefore, anti-CD25-F(ab’)_2_ was specifically delivered and bound to intratumoral CD25^+^ cells, including Tregs *in vivo*.

### CD25-targeted NIR-DPR locally and predominantly decreased intratumoral Treg populations and impaired their function in vivo

**Figure 4.**
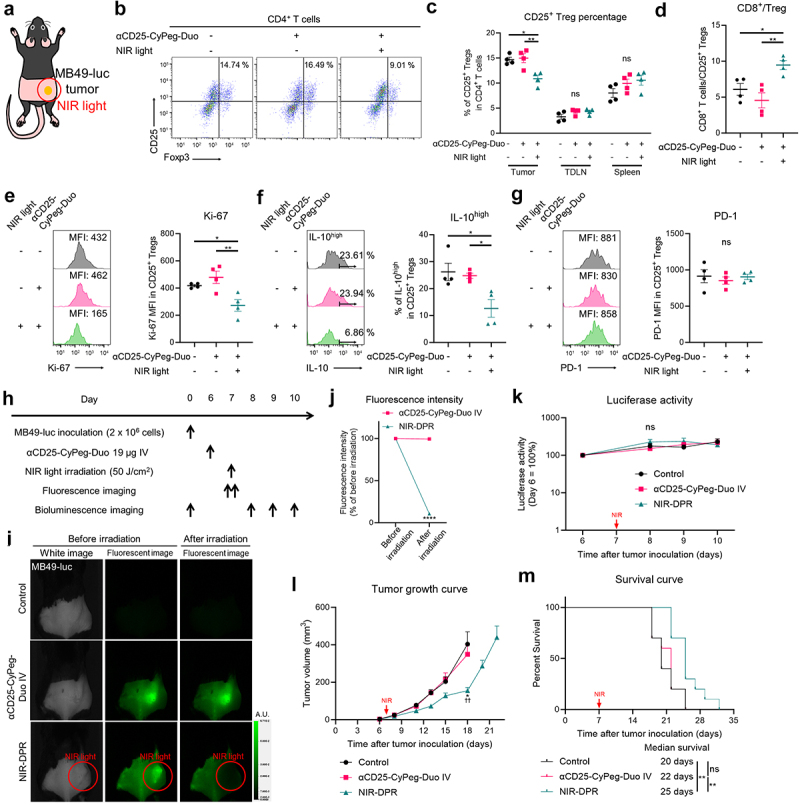
In vivo therapeutic efficacy of CD25-targeted NIR-DPR in a MB49-luc tumor mouse model.

We assessed how CD25-targeted NIR-DPR damaged CD25^+^ Tregs using *in vivo* MB49-luc tumor models. Twenty-four hours after intravenous infusion of αCD25-CyPeg-Duo, NIR light was irradiated only to the tumor as shown in Figure 4a. Twenty-four hours later, the tumor, TDLN, and spleen were harvested and analyzed by flow cytometry. In the tumor, the percentage of CD25^+^ Tregs among CD4^+^ T cells was slightly but significantly decreased after CD25-targeted NIR-DPR (Figure 4b,c). Meanwhile, in the TDLN and spleen, the percentage of CD25^+^ Treg was not significantly changed after CD25-targeted NIR-DPR (Figure 4c). The ratio of CD8^+^ T cells to Tregs, a well-known index of strong anti-cancer immunity,^27^ was significantly increased in the tumor after CD25-targeted NIR-DPR (Figure 4d). Furthermore, CD25-targeted NIR-DPR significantly reduced Ki-67 expression and IL-10 positivity in intratumoral CD25^+^ Tregs (Figure 4e,f). Meanwhile, CD25-targeted NIR-DPR had no effect on PD-1 expression in intratumoral CD25^+^ Tregs (Figure 4g). Since CD25^+^ Th populations were detected in MB49-luc tumors (Figure 4b), we further assessed the effect of CD25-targeted NIR-DPR on intratumoral CD25^+^ Ths. CD25-targeted NIR-DPR did not significantly decrease CD25^+^ Th populations in the tumor (Supplementary Figure S6A). Although it was not statistically significant, CD25-targeted NIR-DPR tended to decrease Ki-67 expression in CD25^+^ Ths (Supplementary Figure S6B), suggesting a minor cytotoxic effect on CD25^+^ Ths. Additionally, only a small percentage of CD25^+^CD8^+^ T cells were detected in MB49-luc tumor and they were not significantly decreased after CD25-targeted NIR-DPR compared to the control (Supplementary Figure S6C). Taken together, CD25- targeted NIR-DPR successfully damaged intratumoral CD25^+^ Treg populations *in vivo* with minimal damage to other immune cell populations.

(a) Diagram of NIR light irradiation. The red circle represents where NIR light was irradiated. (b)–(g) CD25-targeted NIR-DPR decreased the proportion of intratumoral Tregs and impaired their functions. Mice with MB49-luc tumors were treated with CD25-targeted NIR-DPR. The tumors, tumor-draining lymph nodes (TDLNs), and spleens were harvested and analyzed by flow cytometry 24 hours after NIR light irradiation. (b) Representative dot plots of CD4^+^ T cells in the tumor. (c) Comparison of the percentage of CD25^+^ Tregs in CD4^+^ T cells in the tumor, TDLN, and spleen (*n* = 4, mean ± SEM; one-way ANOVA followed by Tukey’s test; *, *p* < 0.05; **, *p* < 0.01; ns, not significant). (d) Comparison of the ratio of CD8^+^ T cells to CD25^+^ Tregs in the tumor (*n* = 4, mean ± SEM; one-way ANOVA followed by Tukey’s test; *, *p* < 0.05; **, *p* < 0.01). (e)–(g) Flow cytometric histograms of Ki-67 (e), IL-10 (f), and PD-1 (g) on intratumoral CD25^+^ Tregs and comparison of their expressions (*n* = 4, mean ± SEM; one-way ANOVA followed by Tukey’s test; *, *p* < 0.05; **, *p* < 0.01; ns, not significant; MFI, mean fluorescence intensity). (h) Treatment schedule. (i) Representative fluorescent imaging at 800 nm before and after treatment in MB49-luc tumor-bearing mice. A.U., arbitrary units. (j) Changes in 800-nm fluorescence intensity at the tumor site before and after treatment (*n* = 10; mean ± SEM; repeated measures two-way ANOVA followed by Sidak’s test; ****, *p* < 0.0001). (k) Luciferase activity after treatment (*n* = 10; mean ± SEM; repeated measures two-way ANOVA followed by Tukey’s test; ns, not significant). (l) Tumor growth curves after treatment (*n* = 10; mean ± SEM; repeated measures two-way ANOVA followed by Tukey’s test; *, *p* < 0.05 vs. the Control group; ^††^, *p* < 0.01 vs. the αCD25-CyPeg-Duo IV group). (m) Survival curves after treatment (*n* = 10, log-rank test with Bonferroni correction; **, *p* < 0.01; ns, not significant).

### In vivo efficacy of CD25-targeted NIR-DPR in syngeneic murine tumor models

We evaluated *in vivo* therapeutic efficacy of CD25-targeted NIR-DPR using MB49-luc tumor models. We treated mice according to the treatment schedule shown in Figure 4h. 800-nm fluorescence was clearly detected at the tumor site prior to NIR light exposure. This fluorescence decreased immediately after NIR light irradiation, representing the photobleaching of CyPeg-Duo (Figure 4i,j). There was no significant difference in the luciferase activity of tumors in the early phase after treatment among the three groups (Figure 4k). However, tumor growth was slightly but significantly slower in mice treated with αCD25-CyPeg-Duo followed by NIR light irradiation compared to the control mice and mice treated with αCD25-CyPeg-Duo only (Figure 4l). In addition, CD25-targeted NIR-DPR slightly improved survival of mice (Figure 4m). Similarly, CD25-targeted NIR-DPR was minimally effective in LL/2-luc tumor models (Supplementary Figure S7). Therefore, CD25-targeted NIR-DPR showed slight but significant therapeutic efficacy in syngeneic murine tumor models.

### In vivo efficacy of CD25-targeted NIR-DPR combined with PD-1 blockade in syngeneic murine tumor models

**Figure 5.**
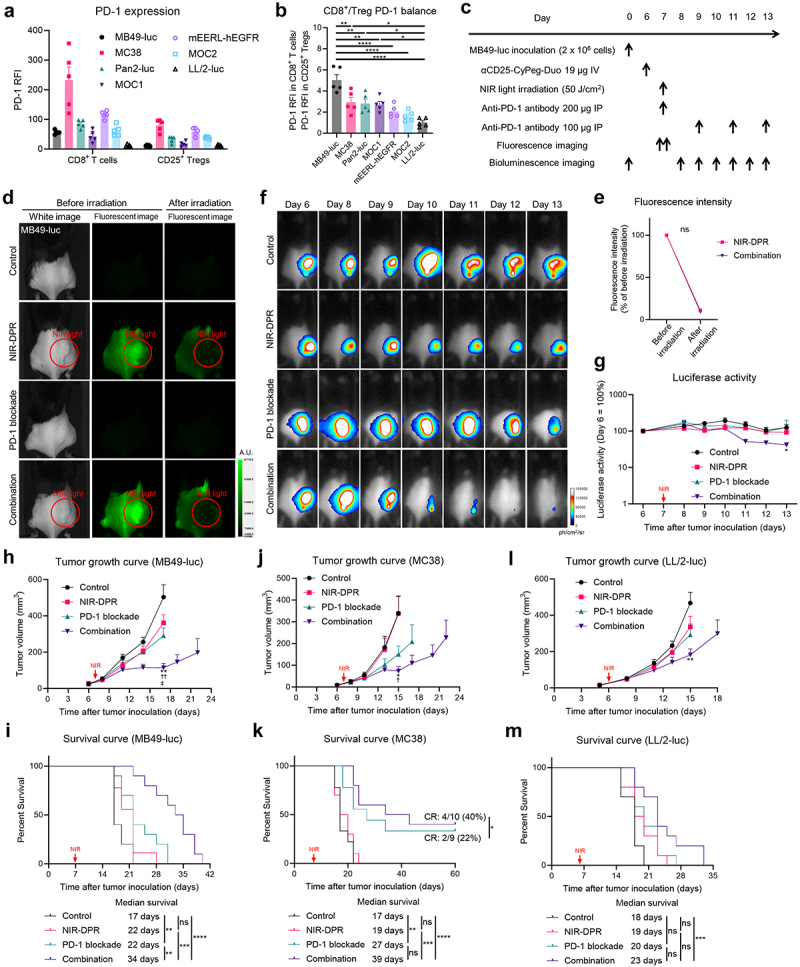
In vivo therapeutic efficacy of CD25-targeted NIR-DPR combined with PD-1 blockade.

PD-1 expression in CD8^+^ T cells and CD25^+^ Tregs was examined using tumor models of MB49-luc, MC38, Pan02-luc, MOC1, mEERL-hEGFR, MOC2, and LL/2-luc. PD-1 expression in CD8^+^ T cells and CD25^+^ Tregs varied widely among the tumor models (Figure 5a). We further evaluated the balance of PD-1 expression between CD8^+^ T cells and CD25^+^ Tregs. MB49-luc tumors showed the highest CD8^+^/Treg PD-1 ratio among the tumor models analyzed, whereas CD8^+^/Treg PD-1 ratio was the lowest in LL/2-luc tumors (Figure 5b). Given the variety of PD-1 expression patterns in intratumoral CD8^+^ T cells and CD25^+^ Tregs, we evaluated the therapeutic efficacy of CD25-targeted NIR-DPR combined with PD-1 blockade using multiple syngeneic murine tumor models. The efficacy of the combination therapy was examined in MB49-luc tumor models, which had the highest CD8^+^/Treg PD-1 ratio (Figure 5c). The 800-nm fluorescence signal at the tumor site was bleached immediately after NIR light irradiation (Figure 5d,e). The luciferase activity at the tumor site was reduced the most in the Combination group. In contrast, the effect of CD25-targeted NIR-DPR alone was minimal. (Figure 5f,g). The Combination group also showed significantly slower tumor growth (Figure 5h) and significantly longer survival compared to the other groups (Figure 5i).

(a) and (b) The balance of PD-1 expression on between CD8^+^ T cells and CD25^+^ Tregs in murine tumors. Tumors were harvested and analyzed by flow cytometry. Relative fluorescence intensity (RFI) was calculated as the ratio of the mean fluorescence intensity of anti-PD-1 antibody to that of the isotype control. (a) PD-1 expression on intratumoral CD8^+^ T cells or CD25^+^ Tregs (*n* = 5, mean ± SEM). (b) Comparison of CD8^+^/Treg PD-1 ratio. CD8^+^/Treg PD-1 ratio was calculated as the ratio of PD-1 RFI in CD8^+^ T cells to PD-1 RFI in CD25^+^ Tregs (*n* = 5, mean ± SEM; one-way ANOVA followed by Tukey’s test; *, *p* < 0.05; **, *p* < 0.01; ****, *p* < 0.0001). (c) Treatment schedule in a MB49-luc tumor model. (d) Representative fluorescent imaging at 800 nm before and after treatment in MB49-luc tumor-bearing mice. A.U., arbitrary units. (e) Changes in 800-nm fluorescence intensity at the tumor site before and after treatment in MB49-luc tumor-bearing mice (*n* = 9–10; mean ± SEM; repeated measures two-way ANOVA followed by Sidak’s test; ns, not significant). (f) Representative bioluminescence images before and after treatment in a MB49-luc tumor model. (g) Luciferase activity after treatment in a MB49-luc tumor model (*n* = 9–10; mean ± SEM; repeated measures two-way ANOVA followed by Sidak’s test; *, *p* < 0.05 vs. the Control group). (h) Tumor growth curves after treatment in a MB49-luc tumor model (*n* = 9–10; mean ± SEM; repeated measures two-way ANOVA followed by Tukey’s test; **, *p* < 0.01 vs. the Control group; ^††^, *p* < 0.01 vs. the NIR-DPR group; ^‡^, *p* < 0.05 vs. the PD-1 blockade group). (i) Survival curves after treatment in a MB49-luc tumor model (*n* = 9–10, log-rank test with Bonferroni correction; **, *p* < 0.01; ***, *p* < 0.001; ****, *p* < 0.0001; ns, not significant). (j) Tumor growth curves after treatment in a MC38 tumor model (*n* = 9–10; mean ± SEM; repeated measures two-way ANOVA followed by Tukey’s test; *, *p* < 0.05 vs. the Control group; ^†^, *p* < 0.05 vs. the NIR-DPR group). (k) Survival curves after treatment in a MC38 tumor model (*n* = 9–10, log-rank test with Bonferroni correction; **, *p* < 0.01; ***, *p* < 0.001; ****, *p* < 0.0001; ns, not significant). In addition, complete remission (CR) rates were compared (Chi-square test; *, *p* < 0.05). (l) Tumor growth curves after treatment in a LL/2-luc tumor model (*n* = 10; mean ± SEM; repeated measures two-way ANOVA followed by Tukey’s test; **, *p* < 0.01 vs. the Control group). (m) Survival curves after treatment in a LL/2-luc tumor model (*n* = 10, log-rank test with Bonferroni correction; ***, *p* < 0.001; ns, not significant).

Next, we analyzed the efficacy of the combination therapy in MC38 tumor models (Supplementary Figure S8A–C), which showed the second highest CD8^+^/Treg PD-1 ratio. The combination therapy showed the most effective inhibition of tumor growth among the treatments tested (Figure 5j). In addition to the longest survival, the Combination group exhibited a CR rate of 40%, which was the highest among the four groups (Figure 5k). The efficacy of the combination therapy was further evaluated in LL/2-luc tumor models (Supplementary Figure S8D–F), which had the lowest CD8^+^/Treg PD-1 ratio. The combination therapy was effective in LL/2-luc tumor models, but it only modestly slowed tumor growth and improved survival compared to the control (Figure 5l,m). Taken together, we concluded that the combination of CD25-targeted NIR-DPR and PD-1 blockade was more effective than either therapy alone, especially in tumors with higher CD8^+^/Treg PD-1 ratio.

### Early host immune responses to CD25-targeted NIR-DPR combined with PD-1 blockade

**Figure 6.**
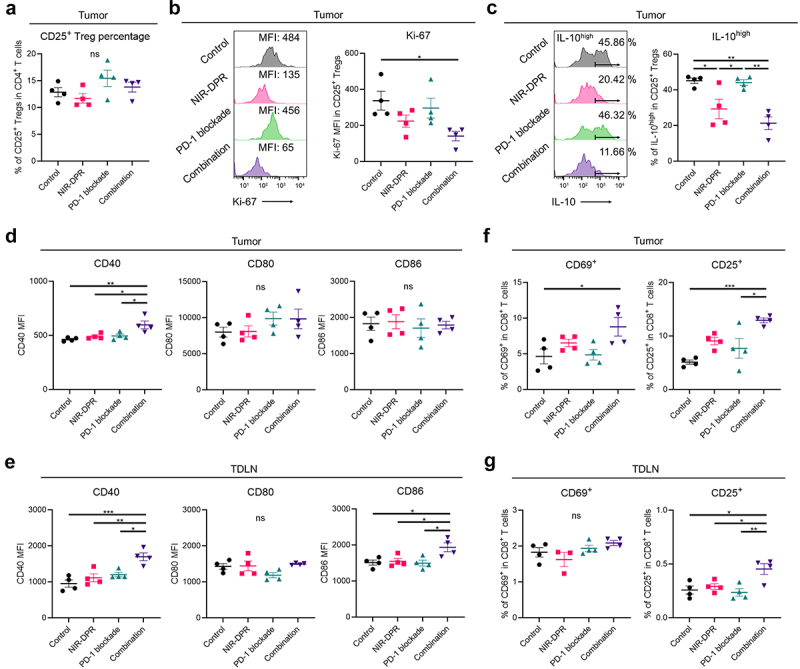
Early host immune responses to CD25-targeted NIR-DPR combined with PD-1 blockade in a MB49-luc tumor mouse model.

To assess early host immune responses to CD25-targeted NIR-DPR combined with PD-1 blockade using MB49-luc tumor models, tumors and TDLNs were harvested and analyzed three days after NIR light irradiation. First, CD25^+^ Treg populations were examined in the tumor. The percentage of CD25^+^ Tregs in CD4^+^ T cells was comparable among the four groups (Figure 6a), suggesting the Treg-depletive effects of CD25-targeted NIR-DPR were transient. Despite this, lower Ki-67 and IL-10 expression in intratumoral CD25^+^ Tregs was observed in the NIR-DPR and Combination groups compared to the Control and PD-1 blockade groups (Figure 6b,c).

Mice with MB49-luc tumors were treated with CD25-targeted NIR-DPR or PD-1 blockade according to the schedule shown in Figure 5c. Immune cell populations in the tumor and tumor-draining lymph node (TDLN) were evaluated by flow cytometry three days after NIR light irradiation. (a) CD25^+^ Treg percentage in the tumor. The percentage of CD25^+^ Tregs in CD45^+^ cells was compared (*n* = 4; mean ± SEM; one-way ANOVA followed by Tukey’s test; ns, not significant). (b) and (c) Expression of functional markers of CD25^+^ Tregs in the tumor. Ki-67 (b) and IL-10 (c) expressions on Tregs were compared (*n* = 4; mean ± SEM; one-way ANOVA followed by Tukey’s test; *, *p* < 0.05; **, *p* < 0.01; MFI, mean fluorescence intensity). (d) and (e) Expression of dendritic cell (DC) maturation markers in the tumor (d) and TDLN (e). CD40, CD80, and CD86 expressions on DCs were compared (*n* = 4; mean ± SEM; one-way ANOVA followed by Tukey’s test; *, *p* < 0.05; **, *p* < 0.01; ***, *p* < 0.001; ns, not significant; MFI, mean fluorescence intensity). (f) and (g) Expression of CD8^+^ T cell activation markers in the tumor (f) and TDLN (g). CD69 and CD25 expressions on CD8^+^ T cells were compared (*n* = 4; mean ± SEM; one-way ANOVA followed by Tukey’s test; *, *p* < 0.05; **, *p* < 0.01; ***, *p* < 0.001; ns, not significant).

Next, DC maturation status was analyzed in the tumor and TDLN. In flow-cytometric analysis of tumors, CD40 expression was significantly increased in the Combination group compared to the other groups (Figure 6d, *left*), but CD80 and CD86 expression was not different among the four groups (Figure 6d, *middle* and *right*). When TDLNs were analyzed, CD40 and CD86 expressions were significantly increased in the Combination group compared to the other groups (Figure 6e, *left* and *right*). CD80 expression was comparable among the four groups (Figure 6e, *middle*). We further assessed the activation status of CD8^+^ T cells. In the analysis of intratumoral CD8^+^ T cell populations, CD69 and CD25 expression was significantly higher in the Combination group compared to the Control group (Figure 6f). In the TDLN, CD25 expression in CD8^+^ T cells was significantly higher in the Combination group compared to the other groups (Figure 6g, *right*). No significant differences were observed in CD69 expression (Figure 6g, *left*), possibly because it is generally upregulated at the very early phase of T cell activation.^28^ These results suggested that the combination therapy synergistically impaired CD25^+^ Treg functioning inducing host immune activation, including DC maturation and CD8^+^ T cell activation.

### CD25-targeted NIR-DPR combined with PD-1 blockade induced marked intratumoral infiltration of cytotoxic CD8^+^ T cells

**Figure 7.**
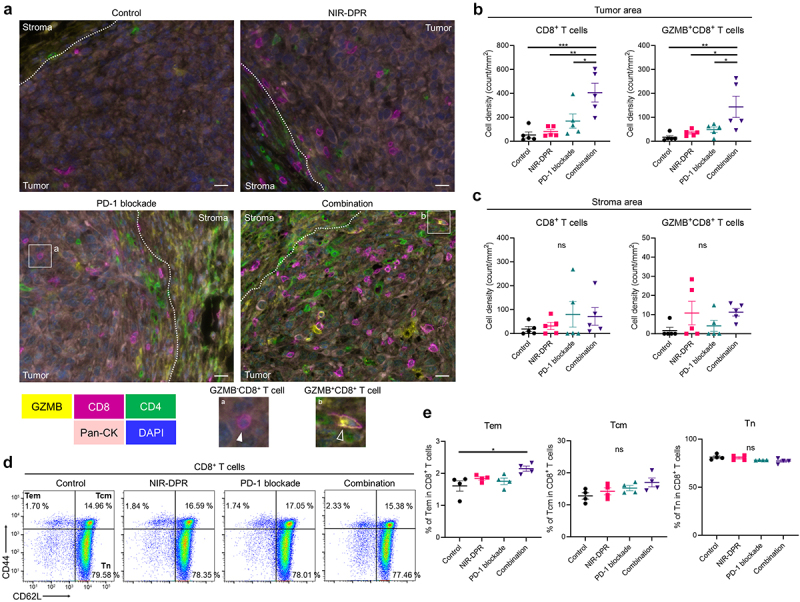
Enhanced anti-cancer immunity was successfully established after CD25-targeted NIR-DPR combined with PD-1 blockade in a MB49-luc tumor mouse model.

To evaluate the intratumoral immune cell infiltration after CD25-targeted NIR-DPR combined with PD-1 blockade, MB49-luc tumors were harvested seven days after NIR light irradiation. Tumors were analyzed by multiplex IHC (Figure 7a). In the tumor area, CD8^+^ T cell density was significantly increased in the Combination group compared to the other groups (Figure 7b, *left*). Furthermore, the density of GZMB^+^CD8^+^ T cells was significantly higher in the Combination group compared to the other groups (Figure 7b, *right*), indicating that the infiltrating CD8^+^ T cells were activated. Meanwhile, in the stromal area, there were no significant differences in CD8^+^ T cell density among the four groups (Figure 7c). These results demonstrate that the combination therapy enhanced intratumoral infiltration of cytotoxic CD8^+^ T cells.

(a)–(c), Tumor-infiltrating CD8^+^ T cells seven days after CD25-targeted NIR-DPR combined with PD-1 blockade in a MB49-luc tumor mouse model. Mice with MB49-luc tumors were treated with CD25-targeted NIR-DPR or PD-1 blockade according to the schedule shown in Figure 5c. Tumors were harvested seven days after NIR light irradiation, and tumor-infiltrating lymphocytes were evaluated by multiplex immunohistochemistry. (a) Representative images (images; ×200; scale bar, 20 μm). Antibody staining of Granzyme B (GZMB), CD8, CD4, and pan-cytokeratin (Pan-CK) is shown in yellow, magenta, green, and pink, respectively. Nucleus are stained with DAPI and shown in blue. The insets (a and b) displayed on the bottom show representative images of a GZMB^−^CD8^+^ T cell (white-filled arrowhead) and GZMB^+^CD8^+^ T cell (white hollow arrowhead). (b) Comparison of CD8^+^ T cell density (*left*) and GZMB^+^CD8^+^ T cell density (*right*) in the tumor area (*n* = 5; mean ± SEM; one-way ANOVA followed with Tukey’s test; *, *p* < 0.05; **, *p* < 0.01; ***, *p* < 0.001). (c) Comparison of CD8^+^ T cell density (*left*) and GZMB^+^CD8^+^ T cell density (*right*) in the stroma area (*n* = 5; mean ± SEM; one-way ANOVA followed with Tukey’s test; ns, not significant). (d)–(e) Effect of CD25-targeted NIR-DPR combined with PD-1 blockade on CD8^+^ memory T cell populations within the tumor-draining lymph node (TDLN). Mice with MB49-luc tumors were treated with CD25-targeted NIR-DPR and PD-1 blockade. The TDLN was harvested 14 days after NIR light irradiation and analyzed by flow cytometry. (d) Representative dot plots of CD8^+^ T cells. CD8^+^ T cells were categorized into effector memory T cell (Tem), central memory T cell (Tcm), and naïve T cell (Tn) based on the expression of CD44 and CD62L. (e) Comparison of each CD8^+^ memory T cell percentage (*n* = 4; mean ± SEM; one-way ANOVA followed by Tukey’s test; *, *p* < 0.05; ns, not significant).

### CD25-targeted NIR-DPR combined with PD-1 blockade significantly induced the differentiation into CD8^+^ memory T cells

To evaluate the differentiation of CD8^+^ memory T cells after CD25-targeted NIR-DPR combined with PD-1 blockade, TDLNs were harvested 14 days after treatment and analyzed by flow cytometry. CD8^+^ T cells were categorized into effector memory T cells (Tem), central memory T cells (Tcm), and naïve T cells (Tn) based on their CD44 and CD62L expressions.^29,30^ The percentage of Tem was significantly higher in the Combination group compared to the Control group (Figure 7d,e, *left*). The percentages of Tcm and Tn were not significantly different among the four groups (Figure 7e, *middle* and *right*). These results suggest that the combination therapy can induce the differentiation of CD8^+^ T cells into Tem.

## Discussion

In this study, we demonstrate a Treg-directed focal therapy that targets CD25^+^ cells and utilizes NIR light to uncage duocarmycin. CD25-targeted NIR-DPR locally and predominantly targets CD25^+^ Tregs in the tumor microenvironment thus eliminating the side effects of systemic Treg ablation. In the *in vivo* tumor models, it successfully decreased intratumoral CD25^+^ Treg populations while impairing their proliferative and immunosuppressive functions. CD25-targeted NIR-DPR as monotherapy slightly but significantly suppressed tumor growth and prolonged survival in syngeneic murine tumor models. However, when CD25-targeted NIR-DPR was combined with PD-1 blockade *in vivo*, it synergistically enhanced the therapeutic efficacy of both agents. The combination therapy provided the best inhibition of tumor growth and the longest survival, especially in tumors with higher CD8^+^/Treg PD-1 ratios. Furthermore, the combination therapy induced significant anti-cancer immune responses, including dendritic cell maturation, increased intratumoral infiltration of cytotoxic CD8^+^ T cells, and increased differentiation into CD8^+^ Tem. Therefore, CD25-targeted NIR-DPR combined with PD-1 blockade has great potential as an anti-cancer combination immunotherapy.

Kumagai et al. has previously shown that the balance of PD-1 expression on CD8^+^ T cells versus Tregs in the tumor microenvironment predicts the clinical efficacy of PD-1 blockade.^14^ Similarly, we have reported that the therapeutic efficacy of PD-1 blockade depends on the balance of CD8^+^ T cells and Tregs in the tumor microenvironment.^15^ We confirmed this by locally depleting CD8^+^ T cells and creating a Treg-dominant tumor microenvironment using near-infrared photoimmunotherapy.^15,31^ PD-1 blockade was effective in CD8^+^ T cell-dominant tumors, whereas it induced hyperprogression in Treg-dominant tumors.^15^ In this study, we demonstrate CD25-targeted NIR-DPR as a new means of reducing Treg cells. Although CD25-targeted NIR-DPR significantly impaired the functions of intratumoral Tregs, its Treg-depletive potential was modest by itself. Nonetheless, our results showed that even a slight shift in the balance of CD8^+^ T cell/Treg in the tumor microenvironment was sufficient to promote the therapeutic effect of PD-1 blockade. Therefore, PD-1 blockade is enhanced when combined with immunomodulatory treatments that partly deplete Tregs.

Several previous studies have shown the anti-cancer efficacy of Treg-directed therapy combined with PD-1 blockade. Anti-CTLA-4 monoclonal antibody combined with PD-1 blockade shows excellent therapeutic outcomes in several solid malignancies.^32,33^ However, it frequently induces serious autoimmune adverse events by systemically depleting Tregs.^7,8^ TGF-β inhibitors and adenosine pathway inhibitors have been developed as Treg-directed therapies.^34–36^ However, these treatments do not directly target Tregs, and non-target pathways might compensate for the inhibited target pathways. Given these limitations of previous Treg-directed therapies, we decided to focally and predominantly kill Tregs using CD25-targeted NIR-DPR. A major strength of NIR-DPR is that it directly targets and impairs Tregs only in the irradiated area and causes no damage to Tregs elsewhere in the body. Thus, NIR-DPR does not cause autoimmune adverse events.

CD25-targeted NIR-DPR synergistically improved the efficacy of PD-1 blockade in various murine tumor models. Therapeutic efficacy of the combination therapy was especially high in MB49-luc tumor models compared to LL/2-luc tumor models. PD-1^low^CD8^+^ T cells were more predominant in LL/2-luc tumors than PD-1^high^CD8^+^ T cells. PD-1 blockade showed no significant efficacy in LL/2-luc tumor models, suggestive of the resistance to PD-1 blockade. Consequently, the efficacy of the combination therapy was significantly higher but modest in LL/2-luc tumor models compared to the control. Such tumors might have immunosuppressive mechanisms other than the PD-1/programmed cell death ligand-1 (PD-L1) axis. Since accumulating evidence shows that there are a wide variety of immunosuppressive mechanisms, including tumor-associated macrophages, myeloid-derived suppressor cells (MDSCs), and T cell exhaustion markers,^37–39^ these mechanisms might be good therapeutic targets in PD-1^low^CD8^+^ T cell-dominant tumors. For example, MDSC-directed therapy showed potent anti-cancer effects in preclinical murine tumor models.^40^

There are several limitations in this study. First, CD25-targeted NIR-DPR could not completely deplete intratumoral CD25^+^ Tregs, and its *in vivo* therapeutic efficacy was modest. We speculate that the modest photonic energy of NIR light limits efficient uncaging reactions.^41^ Further improvement of CyPeg-Duo is necessary to enhance its Treg-depletive potential by increasing the efficiency of payload release in response to NIR light and the number of CyPeg-Duo molecules conjugated with one F(ab’)^2^. Still, CD25-targeted NIR-DPR significantly decreased Ki-67 and IL-10 expression in CD25^+^ Tregs, demonstrating that while it may not have killed Tregs it impaired functioning of intratumoral Tregs. Second, more detailed evaluation of host immune responses to NIR-DPR at multiple timepoints may elucidate the mechanism of the activation of anti-cancer immunity by NIR-DPR. Third, we irradiated NIR light only to tumor sites because we focused on Treg inhibitory function within tumors. However, Tregs can inhibit T cell activation within lymph nodes as well. Thus, NIR-DPR should be tested in the TDLN or both TDLN and tumor as a next step. Fourth, we did not assess the efficacy of CD25-targeted NIR-DPR combined with PD-L1 blockade. Since PD-L1 blockade works by inhibiting the PD-1/PD-L1 axis, our combination strategy would be anticipated to also work with PD-L1 blockade. This should be evaluated in further experiments. Finally, the therapeutic effects of CD25-targeted NIR-DPR were not evaluated using orthotopic tumor models in this study.

In conclusion, we developed a novel local and selective Treg-directed therapy by targeting CD25 and utilizing a NIR photocaging group that uncages duocarmycin upon exposure to NIR light. Our CD25-targeted NIR-DPR partially depletes Tregs only in the target area and impairs their proliferative and immunosuppressive functions. Although its *in vivo* therapeutic efficacy as monotherapy was modest, CD25-targeted NIR-DPR synergistically improved the efficacy of PD-1 blockade in syngeneic murine tumor models. Therefore, CD25-targeted NIR-DPR combined with PD-1 blockade is considered a promising cancer immunotherapy. Given the ease of NIR light application coupled with its deep tissue-penetration of NIR light, this combination therapy has a potential to be widely applicable to clinical settings.

## Supplementary Material

Supplementary Data_Oncoimmunology R1.docx

## Data Availability

The data that support the findings of this study are available from the first or corresponding author, [HF or HK], upon reasonable request.
