# Supplementary material for: Near-infrared duocarmycin photorelease from a Treg-targeted antibody-drug conjugate improves efficacy of PD-1 blockade in syngeneic murine tumor models
Source: Oncoimmunology. 2024 Jun 20;13(1):2370544. doi: 10.1080/2162402X.2024.2370544 (PMC11195482; doi:10.1080/2162402X.2024.2370544)
Supplement: Supplementary Data_Oncoimmunology R1.docx [file KONI_A_2370544_SM1127.docx]

**Supplementary Material**

**Title:** Near-infrared duocarmycin photorelease from a Treg-targeted antibody-drug conjugate improves efficacy of PD-1 blockade in syngeneic murine tumor models

**Authors:** Hiroshi Fukushima^1^, Aki Furusawa^1^, Seiichiro Takao^1^, Ebaston Thankarajan^2^, Michael P Luciano^2^, Syed Muhammad Usama^2^, Makoto Kano^1^, Shuhei Okuyama^1^, Hiroshi Yamamoto^1^, Motofumi Suzuki^1^, Miyu Kano^1^, Peter L Choyke^1^, Martin J Schnermann^2^, Hisataka Kobayashi^1^

**Affiliations:** ^1^Molecular Imaging Branch, Center for Cancer Research, National Cancer Institute, NIH, Bethesda, MD, 20892, USA

^2^Chemical Biology Laboratory, Center for Cancer Research, National Cancer Institute, NIH, Frederick, MD 21702, USA

**Corresponding author:** Hisataka Kobayashi, M.D., Ph.D.

Molecular Imaging Branch, Center for Cancer Research, National Cancer Institute, NIH, 10 Center Drive, Bethesda, MD, 20892, USA

Tel: 240-858-3069; Fax: 240-541-4527; E-mail address: [kobayash@mail.nih.gov](mailto:kobayash@mail.nih.gov)

**List of Supplementary** **material**

Supplementary Methods

Supplementary Figure 1. Synthetic scheme for synthesis of CyPeg-Duo-NHS ester.

Supplementary Figure 2. SDS-PAGE of αCD25-CyPeg-Duo and absorbance and fluorescence spectra for CyPeg-Duo-NHS ester.

Supplementary Figure 3. Gating strategies in flow-cytometric analysis of splenocytes.

Supplementary Figure 4. Gating strategies in flow-cytometric analysis of tumors and tumor-draining lymph nodes (TDLNs).

Supplementary Figure 5. Effect of CD25-targeted NIR-DPR on Ki-67 and IL-10 expressions in CD25^+^ Tregs *ex vivo*.

Supplementary Figure 6. Effect of CD25-targeted NIR-DPR on CD25^+^ Ths and CD25^+^CD8^+^ T cells in the tumor.

Supplementary Figure 7. *In vivo* therapeutic efficacy of CD25-targeted NIR-DPR in a LL/2-luc tumor mouse model.

Supplementary Figure 8. CD25-targeted NIR-DPR combined with PD-1 blockade in MC38 and LL/2-luc tumor mouse models.

Supplementary Table 1. List of key resource identifiers in this study.

NMR and HRMS data of synthesized compounds

References

**Supplementary Methods**

**Synthesis of CyPeg-Duo-NHS ester**

Unless stated otherwise, reactions were conducted in oven- dried glassware under an atmosphere of nitrogen or argon using anhydrous solvents. All commercially obtained reagents were used as received. Duocarmycin DM was purchased from Levena Biopharma (San Diego, CA). Flash column chromatography was performed using reversed phase (100 Å, 20–40 micron particle size, RediSep® Rf Gold® Reversed-phase C18 or C18Aq) on a CombiFlash® Rf 200i (Teledyne Isco, Inc., Lincoln, NE). High-resolution LC/MS analyses were conducted on a Thermo-Fisher LTQ-Orbitrap-XL hybrid mass spectrometer system with an Ion MAX API electrospray ion source. Analytical LC/MS was performed using a Shimadzu LCMS-2020 Single Quadrupole utilizing a Kinetex 2.6 μm C18 100 Å (2.1 x 50 mm) column obtained from Phenomenex, Inc (Torrance, CA). Runs employed a gradient of 0→90% H_2_O/MeCN + 0.1% aqueous formic acid over 4.5 min at a flow rate of 0.2 mL/min.^1^H NMR and ^13^C NMR spectra were recorded on Bruker spectrometers (at 400 or 500 MHz or at 100 or 125 MHz) and are reported relative to deuterated solvent signals. Data for ^1^H NMR spectra are reported as follows: chemical shift (δ ppm), multiplicity, coupling constant (Hz), and integration. Data for ^13^C NMR spectra are reported in terms of chemical shift. Absorbance curves were obtained on a Jasco-V770 spectrophotometer operated by Spectra manager software. Fluorescence traces were recorded on a PTI QuantaMaster steady-state spectrofluorimeter operated by FelixGX 4.2.2 software, with 5 nm excitation and emission slit widths, and a 0.1 s integration rate. Data analyses were performed using GraphPad Prism 10.

Synthesis of **(2)**: Grubbs II catalyst (102 mg, 0.16 mmol), methyl acrylate (1.47 mL, 16.2 mmol) were added to a solution of compound **1** (500 mg, 3.2 mmol) in DCM (4 mL) in a round bottom flask under Ar atmosphere and stirred at 40 °C for 18 h.[1] The reaction mixture was concentrated and directly purified by flash chromatography on (SiO_2_, hexane/ethyl acetate, 3:1) gave (535 mg, 78 %) as an oil. ^1^H NMR (400 MHz, CDCl_3_) δ 7.00 (dt, *J* = 15.7, 4.1 Hz, 1H), 6.12 (dt, *J* = 15.7, 2.1 Hz, 1H), 4.22 (dd, *J* = 4.2, 2.1 Hz, 2H), 3.78 (dq, *J* = 5.5, 2.8 Hz, 1H), 3.75 (s, 3H), 2.59 (ddd, *J* = 15.6, 10.5, 5.8 Hz, 2H), 2.32 – 2.22 (m, 2H), 2.17 – 2.07 (m, 2H), 2.00 – 1.89 (m, 2H). ^13^C NMR (100 MHz, CDCl_3_) δ 210.7, 166.7, 144.7, 120.7, 73.0, 66.9, 51.6, 37.0, 30.4. HRMS (ESI) calculated for C11H16O4 212.1040, observed 212.0704.

Synthesis of **(3)**: To a solution of **2** (110 mg, 0.50 mmol) in [EtOH](https://www.commonorganicchemistry.com/Common_Reagents/Ethanol/Ethanol.htm) (15 mL) was added [10% Pd/C](https://www.commonorganicchemistry.com/Common_Reagents/Palladium_on_Carbon/Palladium_on_Carbon.htm) (11 mg). The system was degassed three times (vacuum/N_2_), then reacted under a H_2_ ballon for 2 h. The mixture was filtered through a pad of celite and the pad was washed with [EtOH](https://www.commonorganicchemistry.com/Common_Reagents/Ethanol/Ethanol.htm) (30 mL). The filtrate was concentrated to provide the product as a clear oil (110 mg, 95%)^1^H NMR (400 MHz, CDCl_3_) δ 3.69 (d, *J* = 2.9 Hz, 1H), 3.67 (s, 3H), 3.53 (t, *J* = 6.1 Hz, 2H), 2.55 (ddd, *J* = 15.7, 10.6, 6.0 Hz, 2H), 2.44 (t, *J* = 7.3 Hz, 2H), 2.24 (dt, *J* = 14.7, 6.0 Hz, 2H), 2.12 – 2.01 (m, 2H), 1.98 – 1.86 (m, 4H). ^13^C NMR (100 MHz, CDCl_3_) δ 211.3, 173.9, 72.8, 67.2, 51.6, 37.1, 30.9, 30.5, 25.4.

Synthesis of **(4)**: At 0 °C, phosphorus oxychloride (38 µL, 0.40 mmol) was added dropwise to anhydrous DMF (73 µL, 2 mmol) at 0°C. After 5 min, compound **3** (22mg, 0.1 mmol) in DMF (1 mL) was added dropwise and the mixture was stirred for 1 h at room temperature. Next, with constant cooling at 0 °C, an aniline/EtOH (1:1 (v/v), 200 µL) mixture was added drop wise. Reaction was continued for an additional 30 min after aniline addition at room temperature, and then the deep purple mixture was poured into ice cold H_2_O/concentrated [HCl](https://www.sciencedirect.com/topics/earth-and-planetary-sciences/hydrochloric-acid) (10:1, 150 mL). A precipitate then was allowed to form for 2 h in an ice bath, which was then filtered, and washed with cold H_2_O (10 mL) and Et_2_O (10 mL), and then dried *in vacuo*: The crude product (27 mg, 60%) was used in this form for the next step. HRMS (ESI) calculated for C25H28ClN_2_O3 439.1785 (M+H), observed 439.1789.

Synthesis of **(6):** To a microwave vial containing indolenine **5** (1.00 g, 3.2 mmol) was added 1-Iodo-2-(2-(2-methoxyethoxy)ethoxy)ethane (4.2 g, 15 mmol) in acetonitrile (3 mL) heated at 120 °C for 24 h.[2] Diethyl ether (10 mL) was charged to vial, and the thick slurry was centrifuged to afford a brown pellet. The crude solid was dissolved in water and purified by reversed-phase chromatography (C18 Aq, 0 to 25% MeCN/water). The solvent was removed *in vacuo* to afford **6** (300 mg, 35 % yield) as a tan solid. ^1^H NMR (400 MHz, DMSO) δ 8.43 (d, *J* = 1.7 Hz, 1H), 8.37 (dd, *J* = 8.8, 5.8 Hz, 2H), 8.16 (d, *J* = 8.9 Hz, 1H), 7.97 (dd, *J* = 8.8, 1.7 Hz, 1H), 4.85 (t, *J* = 5.0 Hz, 2H), 3.95 (t, *J* = 4.9 Hz, 2H), 3.53 – 3.48 (m, 2H), 3.24 (dd, *J* = 5.9, 3.6 Hz, 2H), 3.12 (s, 2H), 2.91 (d, *J* = 7.7 Hz, 2H), 2.68 (p, *J* = 1.8 Hz, 1H), 2.34 (p, *J* = 1.9 Hz, 1H), 1.77 (s, 6H). ^13^C NMR (101 MHz, DMSO) δ 198.7, 147.3, 139.3, 137.1, 133.0, 131.7, 127.4, 126.9, 126.1, 123.6, 114.2, 71.5, 70.3, 70.2, 69.8, 67.3, 58.4, 56.0, 48.5, 40.5, 21.9, 14.7. HRMS (ESI) calculated for C22H30NO6S 436.1788 (M+H), observed 436.1792.

Synthesis of **(7):** To a microwave vial, indolenine **6** (100 mg, 0.22 mmol) and chloride **4** (55 mg, 0.06 mmol). ethanol (2.5 mL), triethylamine (810 μL, 0.51 mmol), and acetic anhydride (109 μL, 1.2 mmol) were added in succession. The yellow solution was heated to 110 °C for 20 min, during which time the reaction transitioned to a deep green color. The reaction was cooled and precipitated in ether. The supernatant was decanted, and the crude solid was dissolved in water (10 mL) and purified by reversed-phase chromatography (C18 Aq, 0 to 30 % MeCN/water). The product-containing fractions were lyophilized to afford **7** (10 mg, 15 % yield) as green solid. ^1^H NMR (400 MHz, MeOD) δ 8.61 (d, *J* = 14.2 Hz, 2H), 8.46 (d, *J* = 1.9 Hz, 2H), 8.37 (d, *J* = 9.0 Hz, 2H), 8.15 – 8.03 (m, 4H), 7.75 (d, *J* = 8.9 Hz, 2H), 6.52 (d, *J* = 14.2 Hz, 2H), 4.58 (t, *J* = 5.1 Hz, 4H), 4.00 (q, *J* = 5.9 Hz, 5H), 3.68 (d, *J* = 9.8 Hz, 5H), 3.64 – 3.60 (m, 4H), 3.56 – 3.51 (m, 4H), 3.46 – 3.41 (m, 4H), 3.32 – 3.28 (m, 4H), 3.22 (s, 6H), 3.07 – 2.98 (m, 2H), 2.84 (dd, *J* = 15.5, 7.0 Hz, 2H), 2.45 (t, *J* = 7.3 Hz, 2H), 2.07 (d, *J* = 2.1 Hz, 12H), 1.96 – 1.88 (m, 2H). ^13^C NMR (125 MHz, MeOD) δ 175.39, 174.27, 148.45, 143.98, 141.51, 141.36, 133.86, 131.13, 131.10, 128.30, 127.05, 124.77, 123.92, 122.41, 112.31, 101.77, 71.70, 71.47, 70.71, 70.28, 69.97, 68.05, 67.26, 57.64, 56.41, 56.24, 56.07, 55.90, 51.10, 50.67, 48.21, 44.74, 31.25, 30.37, 26.54, 25.18. HRMS (ESI) calculated for C57H72ClN2O15S2 1123.4057, observed 1123.4048

Synthesis of **(8):** To a microwave vial, chloride **7** (15 mg, 0.013 mmol) and DMF (1 mL). *N*,*N*′- diethylethylene diamine (8 μL, 0.05 mmol) and diisopropylethylamine (7.0 μL, 0.040 mmol) were added and the reaction was heated to 110 °C for 20 min, during which time the reaction color transitioned from green to dark blue. Then boc-anhydride (29 mg, 0.13 mmol) was added to the solution and stirred for 1h at room temperature. The reaction was cooled and diluted with H_2_O (8 mL), and the solution was directly purified by reversed-phase chromatography (C18 gold column, 0 to 30% MeCN/water). The product-containing fractions were lyophilized to afford **8** (8 mg, 48% yield) as bluish-green solid. ^1^H NMR (400 MHz, MeOD) δ 8.43 (d, *J* = 1.8 Hz, 2H), 8.32 (d, *J* = 9.0 Hz, 2H), 8.06 (d, *J* = 8.9 Hz, 2H), 8.02 (dd, *J* = 9.0, 1.8 Hz, 2H), 7.94 (d, *J* = 13.7 Hz, 2H), 7.67 (d, *J* = 8.9 Hz, 2H), 6.25 (d, *J* = 13.7 Hz, 2H), 4.44 (d, *J* = 6.1 Hz, 4H), 3.97 (t, *J* = 5.1 Hz, 4H), 3.94 – 3.80 (m, 5H), 3.70 – 3.50 (m, 15H), 3.48 – 3.41 (m, 4H), 3.32 – 3.29 (m, 4H), 3.23 (s, 6H), 2.88 (d, *J* = 14.7 Hz, 2H), 2.71 (s, 2H), 2.43 (t, *J* = 7.3 Hz, 2H), 2.03 (d, *J* = 1.9 Hz, 12H), 1.90 (q, *J* = 6.7 Hz, 3H), 1.47 (m, 12H), 1.16 (t, *J* = 7.0 Hz, 3H). ^13^C NMR (125 MHz, MeOD) δ 174.1, 172.6, 143.1, 142.0, 140.7, 132.4, 130.9, 130.4, 128.5, 127.0, 124.5, 122.1, 112.1, 97.5, 79.9, 71.5, 70.8, 70.3, 70.0, 67.9, 67.1, 57.7, 56.4, 56.2, 56.1, 55.9, 50.6, 50.0, 48.2, 44.0, 42.6, 30.5, 30.4, 27.7, 27.7, 27.4, 25.2, 14.1, 12.9.

Synthesis of **(9):** To a microwave vial at 0 °C, **8** (5 mg) and MeOH: H_2_O (1 mL, 1:1) and 1M NaOH (200 μL) were added. The reaction was allowed to warm to rt over 2 h. LC-MS indicated 100 % deprotection of methyl ester. The solvent was evaporated under N_2_ and TFA 0.5 mL added and stirred for 30 min under an argon atmosphere. The TFA was evaporated under N_2_, the mixture redissolved in 1mL dry DMF, filtered and then precipitated with Et_2_O (5 mL) to obtained **9** (3 mg), which was used in the next step without purification.

Synthesis of **(11):** To a microwave vial duocarmycin DM (1.9 mg, 0.0032 mmol) and MeCN (0.2 mL). Diisopropylethylamine (1.4 μL, 0.0075 mmol) and a solution of 4-nitrophenylchloroformate (1 mg, 0.005 mmol) in MeCN (0.25 mL) were added in succession. The clear, light yellow solution was stirred for 40 min at room temperature, after which time HPLC indicated 80 % conversion to mixed carbonate **10**. In separate vessel **9** (2.6 mg, 0.004 mmol) was dissolved in DMF (0.5 mL) under argon, to which diisopropylethylamine (1 μL, 0.004 mmol) was added. This DMF solution was combined with mixed carbonate **10**, and the dark blue mixture was heated to 65 °C for 60 min. The reaction was cooled and diluted with H_2_O (7 mL), and the solution was directly purified by reversed-phase chromatography (C18 gold column, 0 to 35 % MeCN/water). The product-containing fractions were lyophilized to afford **11** (1 mg, 30 % yield) as a bluish- green solid. LCAP at 250 nm: 95%; HRMS (ESI) calculated for C_89_H_109_ClN_7_O_19_S_2_ 1678.6903 observed 1680.69034 (M+2H) and 840.35164 (M+2H)+2

Synthesis of **(****CyPeg-Duo-NHS ester):** To a microwave vial, **11** (1.0 mg, 0.0006 mmol) and *N*,*N*,*N*′,*N*′-tetramethyl-*O*-(*N*- succinimidyl)uronium tetrafluoroborate (0.2 mg, 0.0012 mmol). DMF (0.5 mL) was added to the vial, followed by *N,N*-diisopropylethylamine (0.2 μL, 0.0012 mmol). The deep blue solution was stirred for 30 min at 25 °C, at which time LC/MS indicated consumption of **17**. The reaction was precipitated into diethyl ether (1.0 mL). The fine suspension was centrifuged, the supernatant decanted, and the pellet resuspended in ethyl acetate (0.5 mL). The procedure was then repeated twice with diethyl ether, and the pellet was placed under vacuum (< 0.1 Torr) for 1 h to afford **CyPeg-Duo-NHS ester** (1 mg, 97 % yield) as a dark blue solid. HRMS (ESI) calculated for C_93_H_112_ClN_8_O_21_S_2_ 1775.7066, observed. 1777.70360 (M+2H) and 888.85721 (M+2H)+2

**Sodium dodecyl sulfate-polyacrylamide gel electrophoresis (SDS-PAGE)**

Conjugation of αCD25-CyPeg-Duo was verified by SDS-PAGE with a 4−12 % gradient polyacrylamide gel (Life Technologies, Gaithersburg, MD, USA; Supplementary Fig. 2A). Unconjugated F(ab’)_2_ was used as a control. After electrophoresis at 80 V for 2.5 h, the gel was imaged with a Pearl Imager (LI-COR Biosciences, Lincoln, NE, USA) using the 800-nm fluorescence channel. The gel was then stained with Colloidal Blue to compare the molecular weight of the conjugate to that of unconjugated F(ab’)_2_.

***In vitro* CD25 expression analysis**

To assess *in vitro* CD25 expression on HT2-A5E and EL4 cells, 2 × 10^5^ cells were incubated with anti-mouse CD25 antibody (clone 3C7; Biolegend, San Diego, CA, USA) or its mouse IgG2b, κ isotype control (clone RTK4530; Biolegend) as well as Fixable Viability Dye (Thermo Fisher Scientific, Rockford, IL, USA) for 30 minutes at 4 °C. The fluorescence of the cells was analyzed by BD FACSLyric (BD Biosciences, San Jose, CA, USA) and FlowJo software (FlowJo LLC, Ashland, OR, USA).

***In vitro* αCD25-CyPeg-Duo cell binding assay**

Cells (1 × 10^5^) were incubated in 100 μL of culture medium containing αCD25-CyPeg-Duo (1.9 μg/mL) for 30 minutes at 4 °C. After washing with phosphate buffered saline (PBS), the fluorescence of the cells was analyzed by BD FACSLyric (BD Biosciences) and FlowJo software (FlowJo LLC). To validate the specific binding of αCD25-CyPeg-Duo, ten-fold molar excess of unconjugated anti-CD25 F(ab’)_2_ was added 30 minutes before the incubation with αCD25-CyPeg-Duo. Dead cells were excluded from the analysis based on the staining with Fixable Viability Dye (Thermo Fisher Scientific).

***In vivo* fluorescence imaging**

Tumor-bearing mice were injected with αCD25-CyPeg-Duo (19 µg) via lateral tail vein. Given the absorbance spectra for CyPeg-Duo (Supplementary Fig. 2B), serial dorsal fluorescence images were obtained with the 800-nm fluorescence channel of a Pearl Imager (LI-COR Bioscience). The images were analyzed with Pearl Cam Software (LI-COR Bioscience). Regions of interest (ROIs) were drawn on the tumor and the non-tumoral region of the contralateral side. Target-to-background ratio (TBR) was calculated as (Mean fluorescence intensity of the tumor)/(Mean fluorescence intensity of the non-tumoral region of the contralateral side).

***Ex vivo* NIR-DPR**

Spleens were extracted from non-tumor bearing mice and single cell suspensions were prepared. Red blood cells (RBCs) were removed by incubating with RBC lysis buffer (BioLegend, San Diego, CA, USA). Splenocytes (1 × 10^7^) were incubated in 500 μL of culture medium containing αCD25-CyPeg-Duo (1.9 μg/mL) for six hours at 37 °C. NIR laser-light (780 nm, 50 J/cm^2^, 150 mW/cm^2^) was applied using an ML6600 laser system (Modulight). Twenty-four hours later, cells were analyzed using FACSLyric (BD Biosciences) and Flowjo software (FlowJo LLC). Dead cells were gated out by Fixable Viability Dye (Thermo Fisher Scientific).

**Supplementary Figure 1**

**Supplementary Figure 1.** Synthetic scheme for synthesis of CyPeg-Duo-NHS ester.

**Supplementary Figure 2**


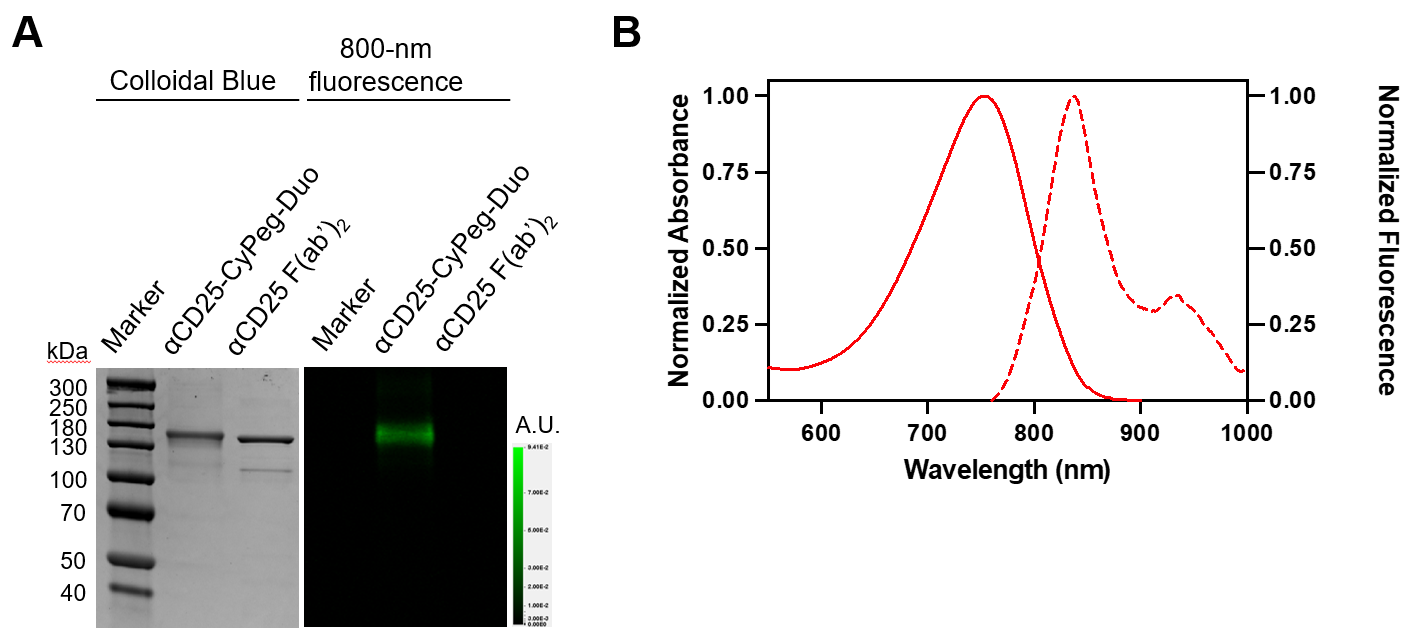


**Supplementary Figure 2** SDS-PAGE of αCD25-CyPeg-Duo and absorbance and fluorescence spectra for CyPeg-Duo-NHS ester.

A, SDS-PAGE of αCD25-CyPeg-Duo (left, colloidal blue staining; right, 800-nm fluorescence). Unconjugated anti-CD25 F(ab’)_2_ was used as a control. αCD25-CyPeg-Duo had the same approximate molecular weight as unconjugated anti-CD25 F(ab’)_2_ but only αCD25-CyPeg-Duo exhibited 800-nm fluorescence. A.U., arbitrary units. B, Normalized absorbance and fluorescence spectra for CyPeg-Duo-NHS ester. Solid and dashed lines show normalized absorbance and fluorescence spectra for CyPeg-Duo-NHS ester, respectively.

**Supplementary Figure 3**

**
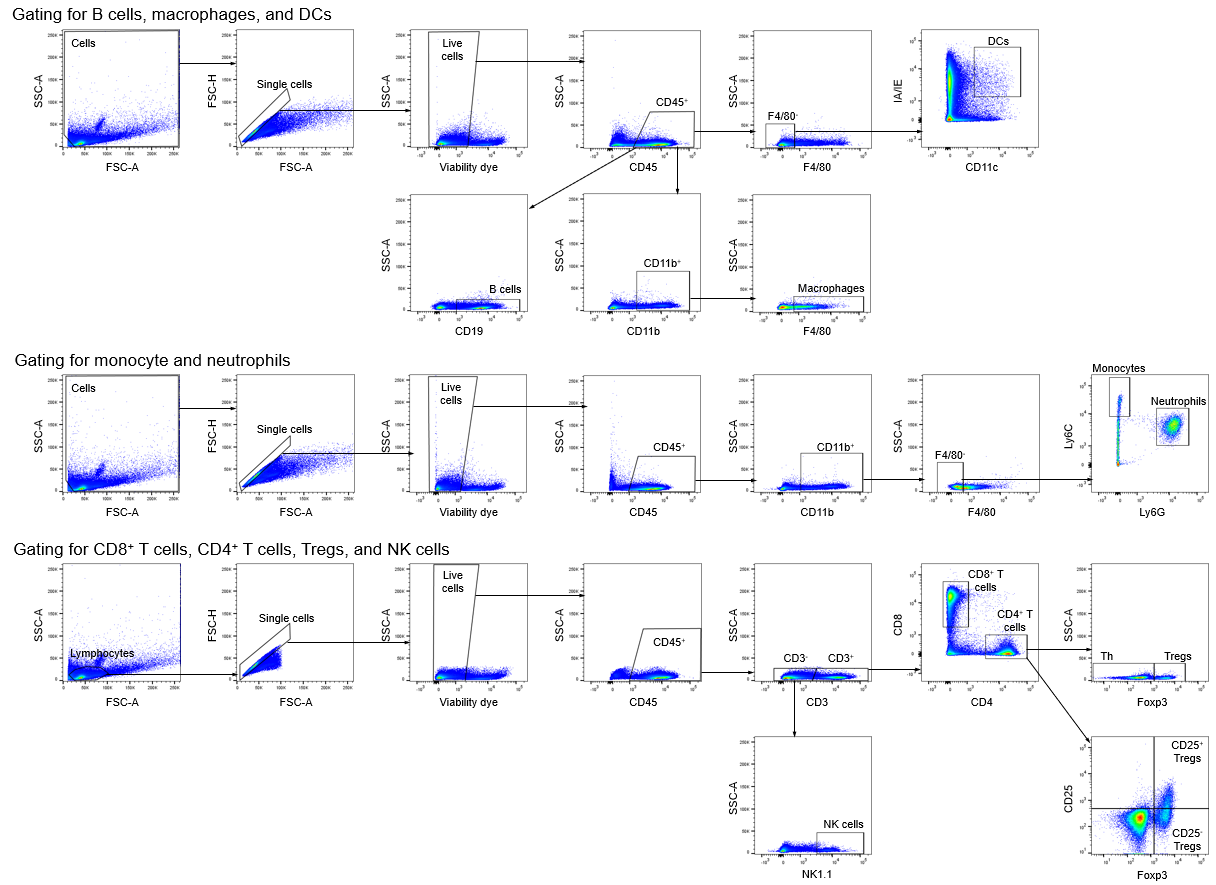
**

**Supplementary Figure 3.** Gating strategies in flow-cytometric analysis of splenocytes.

DC, dendritic cell; NK, natural killer; Th, helper T cell; Treg, regulatory T cell.

**Supplementary Figure 4**

**
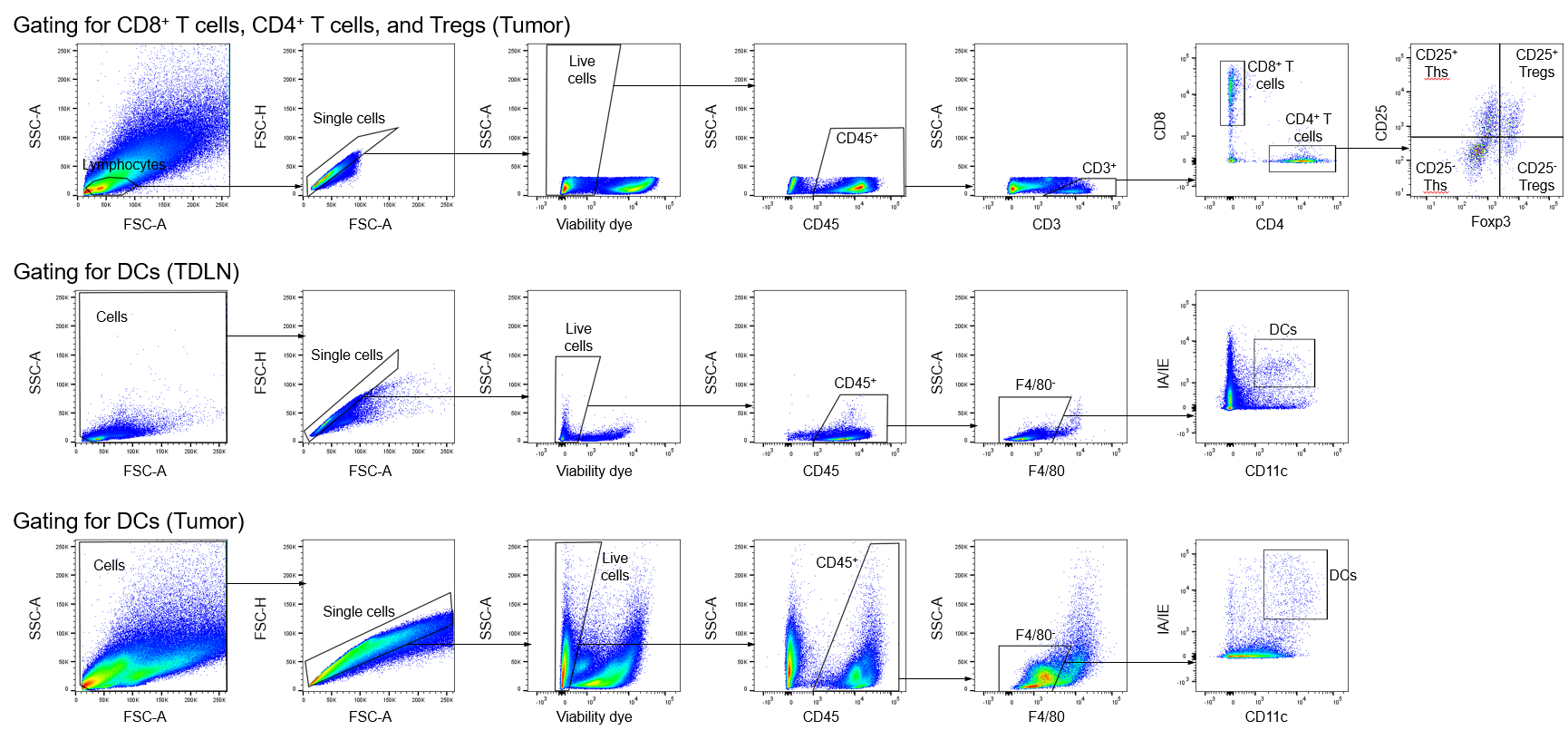

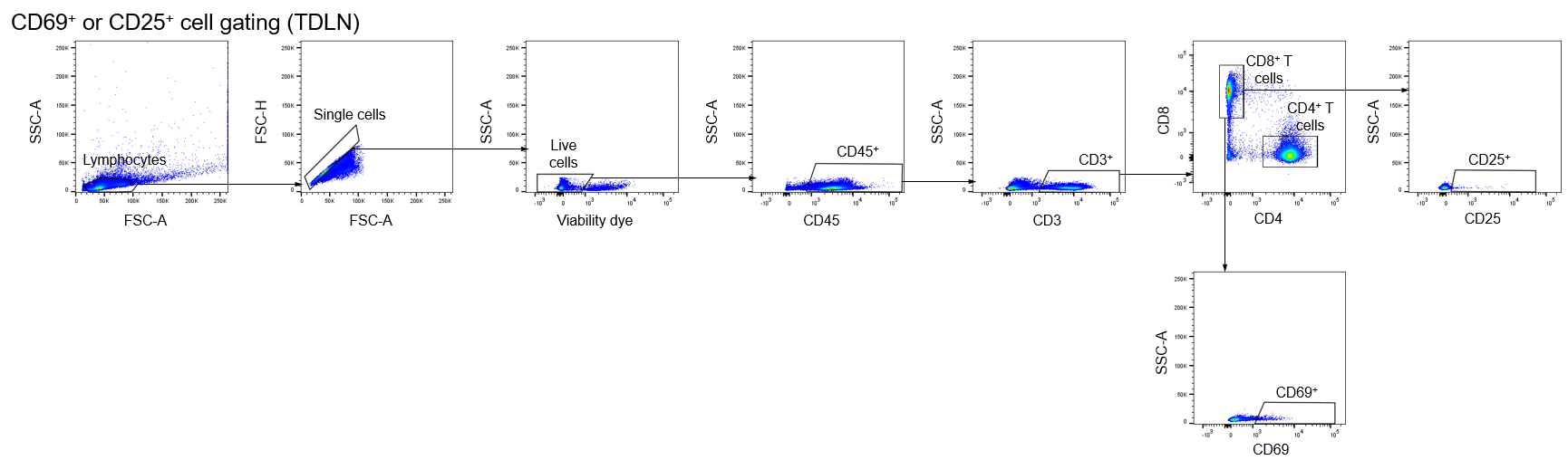

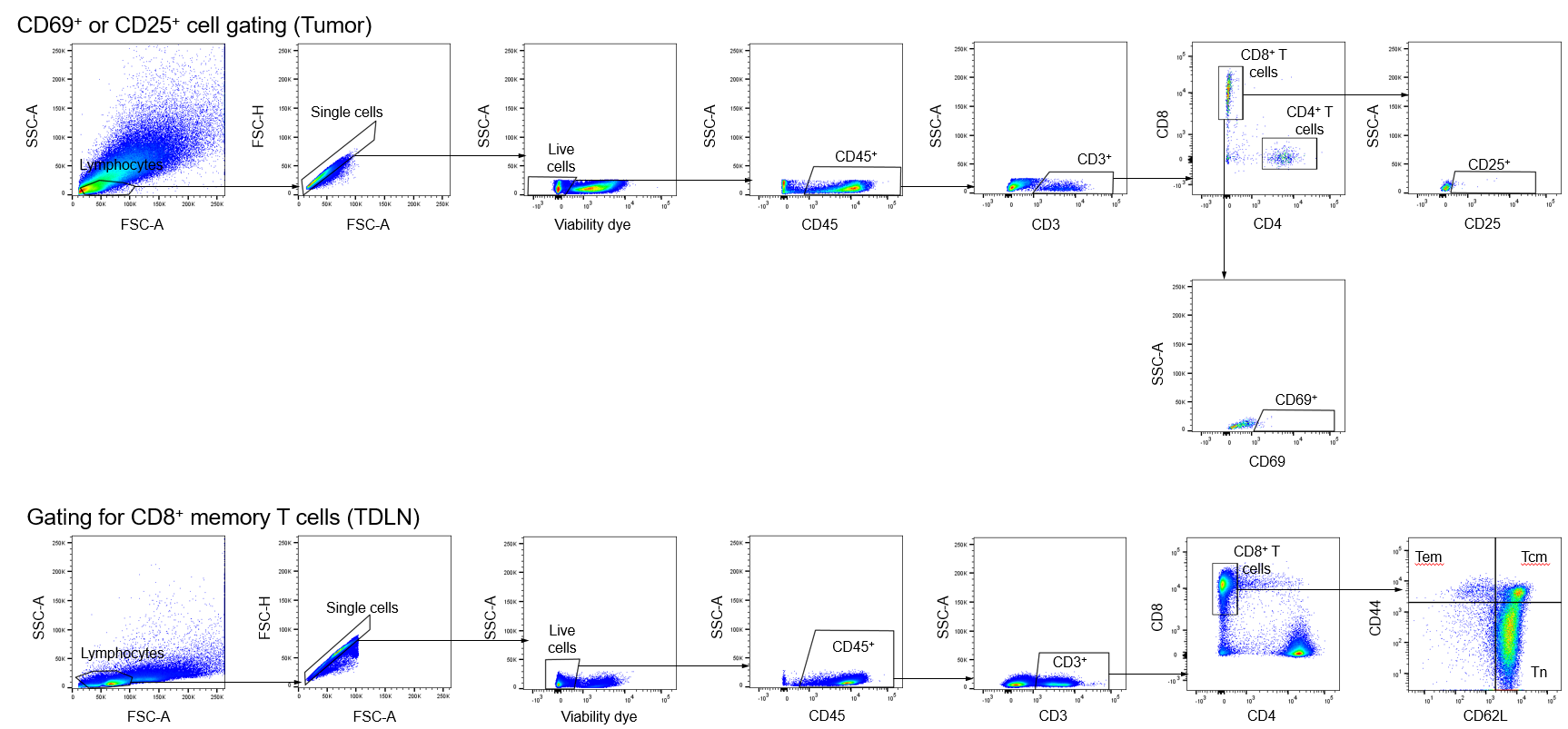
**

**Supplementary Figure 4.** Gating strategies in flow-cytometric analysis of tumors and tumor-draining lymph nodes (TDLNs).

DC, dendritic cell; Th, helper T cell; Treg, regulatory T cell; Tem, effector memory T cell; Tcm, central memory T cell; Tn, naïve T cell.

**Supplementary Figure 5**

**
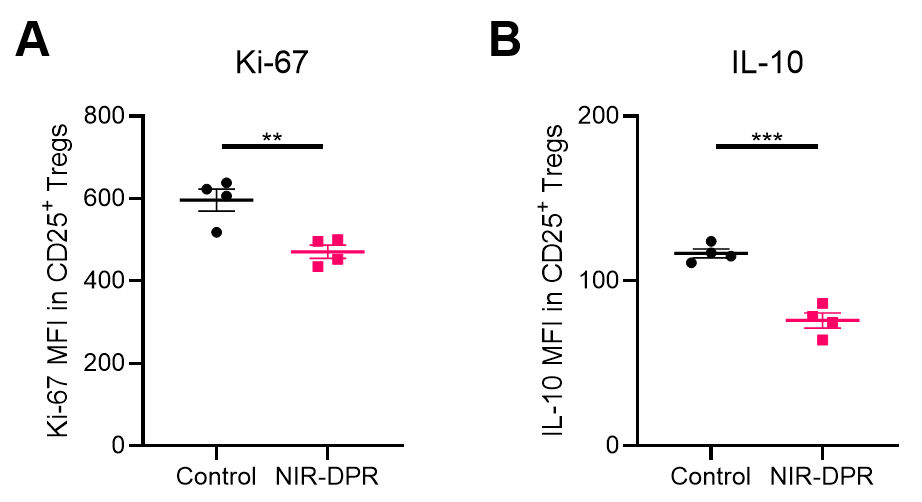
**

**Supplementary Figure 5.** Effect of CD25-targeted NIR-DPR on Ki-67 and IL-10 expressions in CD25^+^ Tregs *ex vivo*.

*Ex vivo* CD25-targeted NIR-DPR for splenocytes was performed. A, Comparison of Ki-67 expression in CD25^+^ Tregs (n = 4, mean ± SEM; unpaired t-test; **, p < 0.01; MFI, mean fluorescence intensity). B, Comparison of IL-10 expression in CD25^+^ Tregs (n = 4, mean ± SEM; unpaired t-test; ***, p < 0.001; MFI, mean fluorescence intensity).

**Supplementary Figure 6**

**
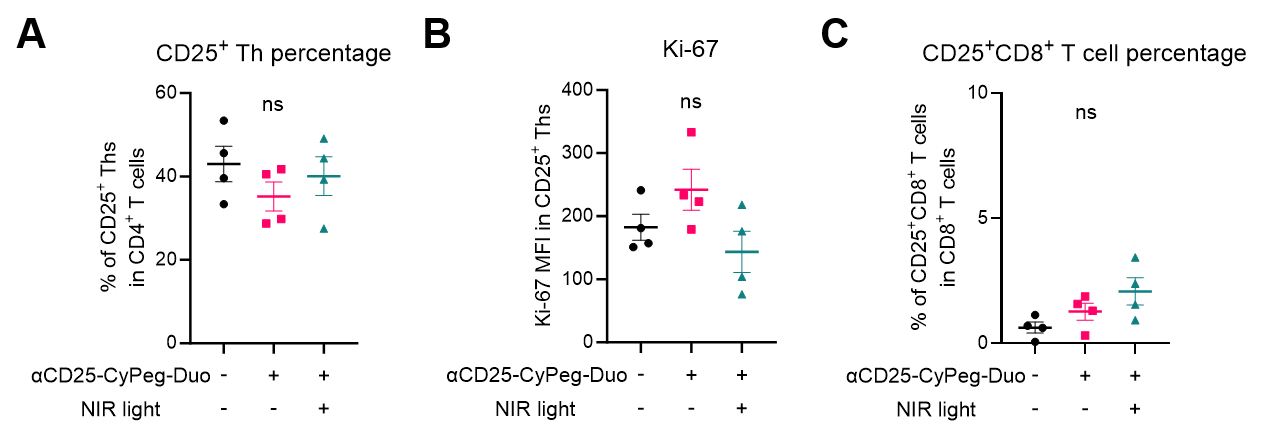
**

**Supplementary Figure 6.** Effect of CD25-targeted NIR-DPR on CD25^+^ Ths and CD25^+^CD8^+^ T cells in the tumor.

Mice with MB49-luc tumors were treated with CD25-targeted NIR-DPR. The tumor was harvested and analyzed by flow cytometry 24 hours after NIR light irradiation. A, Comparison of the percentage of CD25^+^ Ths in CD4^+^ T cells in the tumor (n = 4, mean ± SEM; one-way ANOVA followed by Tukey’s test; ns, not significant). B, Comparison of Ki-67 expression in CD25^+^ Ths in the tumor (n = 4, mean ± SEM; one-way ANOVA followed by Tukey’s test; ns, not significant; MFI, mean fluorescence intensity). C, Comparison of the percentage of CD25^+^CD8^+^ T cells in CD8^+^ T cells in the tumor (n = 4, mean ± SEM; one-way ANOVA followed by Tukey’s test; ns, not significant).

**Supplementary Figure 7**

**
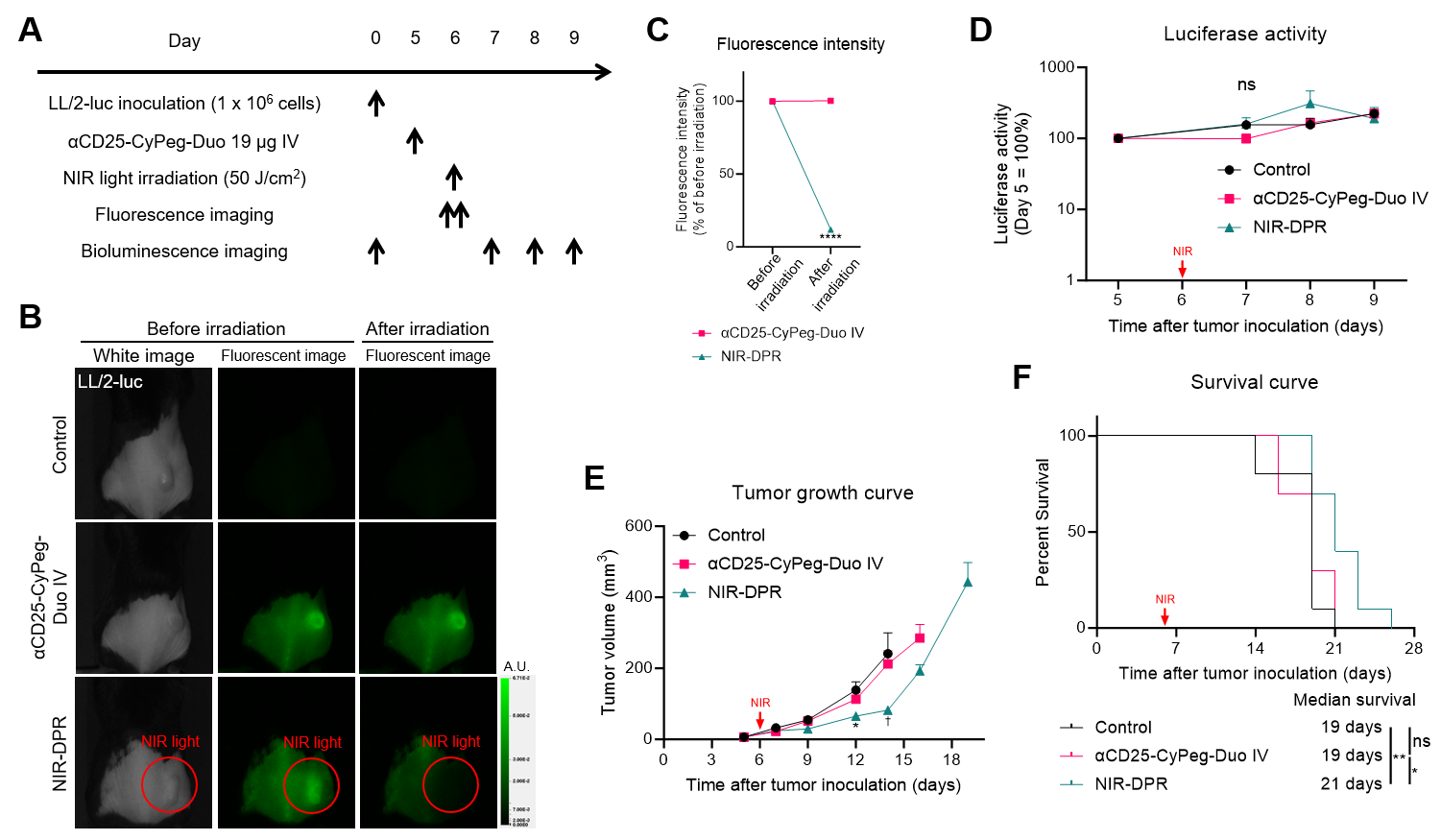
**

**Supplementary Figure 7.** *In vivo* therapeutic efficacy of CD25-targeted NIR-DPR in a LL/2-luc tumor mouse model.

A, Treatment schedule of CD25-targeted NIR-DPR. B, Representative fluorescent imaging at 800 nm before and after treatment in LL/2-luc tumor-bearing mice. A.U., arbitrary units. C, Changes in 800-nm fluorescence intensity at the tumor site before and after treatment (n = 10; mean ± SEM; repeated measures two-way ANOVA followed by Sidak’s test; ****, p < 0.0001). D, Luciferase activity after treatment (n = 10; mean ± SEM; repeated measures two-way ANOVA followed by Tukey’s test; ns, not significant). E, Tumor growth curves after treatment (n = 10; mean ± SEM; repeated measures two-way ANOVA followed by Tukey's test; *, p < 0.05 vs. the Control group; †, p < 0.05 vs. the αCD25-CyPeg-Duo IV group). F, Survival curves after treatment (n = 10, log-rank test with Bonferroni correction; *, p <0.05; **, p < 0.01; ns, not significant).

**Supplementary Figure 8**


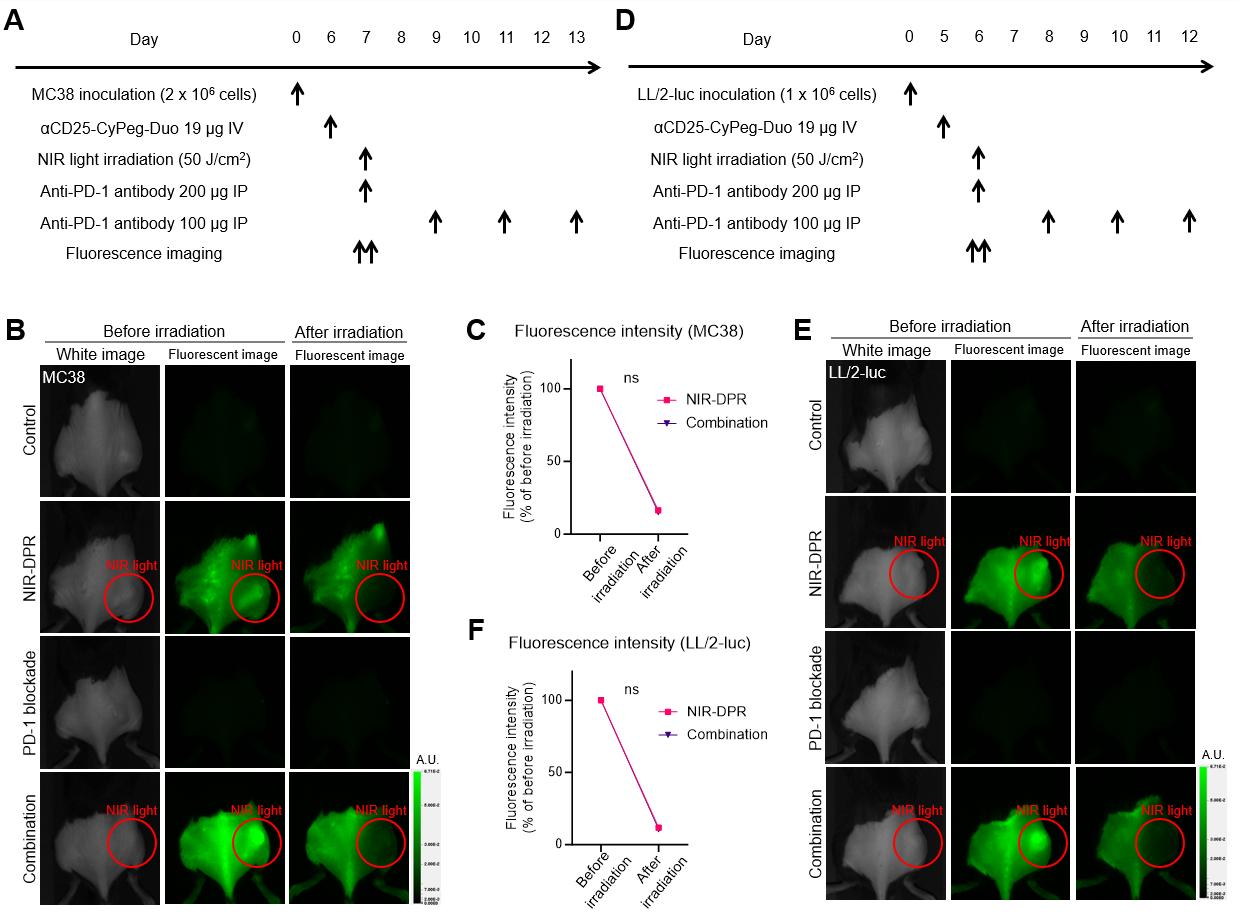


**Supplementary Figure 8.** CD25-targeted NIR-DPR combined with PD-1 blockade in MC38 and LL/2-luc tumor mouse models.

A, Treatment schedule in a MC38 tumor mouse model. B, Representative fluorescent imaging at 800 nm before and after treatment in MC38 tumor-bearing mice. A.U., arbitrary units. C, Changes in 800-nm fluorescence intensity at the tumor site before and after treatment in MC38 tumor-bearing mice (n = 9–10; mean ± SEM; repeated measures two-way ANOVA followed by Sidak’s test; ns, not significant). D, Treatment schedule in a LL/2-luc tumor mouse model. E, Representative fluorescent imaging at 800 nm before and after treatment in LL/2-luc tumor-bearing mice. A.U., arbitrary units. F, Changes in 800-nm fluorescence intensity at the tumor site before and after treatment in LL/2-luc tumor-bearing mice (n = 10; mean ± SEM; repeated measures two-way ANOVA followed by Sidak’s test; ns, not significant).

**Supplementary Table 1.** List of key resource identifiers in this study.

| Item | Vendor | RRID |
| --- | --- | --- |
| Anti-mouse CD25 antibody | Bio X Cell | AB_1107619 |
| HT2-A5E | ATCC | CVCL_0319 |
| EL4 | ATCC | CVCL_0255 |
| MB49-luc | GenTarget Inc | CVCL_7076 |
| LL/2-luc | Imanis Life Sciences | CVCL_A4CM |
| MC38 | Dr. Thomas Waldmann, NIH | CVCL_B288 |
| MOC1 | Kerafast | CVCL_ZD32 |
| MOC2 | Kerafast | CVCL_ZD33 |
| C57BL/6 mice | Jackson Laboratory | IMSR_JAX: 000664 |
| Anti-CD8 alpha antibody [EPR20305] | Abcam | AB_2860566 |
| Anti-CD4 antibody [EPR19514] | Abcam | AB_2686917 |
| Anti-pan-cytokeratin polyclonal antibody | Bioss | AB_10855057 |
| Anti-Foxp3 antibody | Novus Biologicals | AB_2929004 |
| Granzyme B antibody | Abcam | AB_304251 |
| Anti-CD11b antibody [EPR1344] | Abcam | AB_2650514 |
| Digoxigenin Recombinant Rabbit Monoclonal Antibody (9H27L19) | Thermo Fisher Scientific | AB_2532342 |
| inForm software | inForm software | SCR_019155 |
| FACSLyric | BD Biosciences | SCR_000401 |
| FlowJo software | FlowJo LLC | SCR_008520 |
| GraphPad Prism | GraphPad software | SCR_002798 |
| FITC anti-mouse CD3e antibody | Biolegend | AB_312671 |
| PerCP/Cyanine5.5 anti-mouse CD3e antibody | Biolegend | AB_893318 |
| Brilliant Violet 510 anti-mouse CD3e antibody | Biolegend | AB_2565879 |
| PE/Cyanine7 anti-mouse CD4 antibody | Biolegend | AB_312729 |
| CD4 Monoclonal Antibody, eFluor 450 | Thermo Fisher Scientific | AB_1272194 |
| Brilliant Violet 510 anti-mouse CD4 antibody | Biolegend | AB_2562608 |
| CD8α Monoclonal Antibody (53-6.7), FITC | Thermo Fisher Scientific | AB_464916 |
| CD8α Monoclonal Antibody, PerCP-Cyanine5.5 | Thermo Fisher Scientific | AB_1107004 |
| CD8α Monoclonal Antibody, APC | Thermo Fisher Scientific | AB_469334 |
| FITC anti-mouse/human CD11b antibody | Biolegend | AB_312789 |
| Pacific Blue anti-mouse/human CD11b Antibody | Biolegend | AB_755986 |
| Brilliant Violet 510 anti-mouse/human CD11b antibody | Biolegend | AB_2629529 |
| FITC anti-mouse CD11c antibody | Biolegend | AB_313775 |
| CD19 Monoclonal Antibody, PerCP-Cyanine5.5 | Thermo Fisher Scientific | AB_1106999 |
| Brilliant Violet 510 anti-mouse CD19 antibody | Biolegend | AB_2562137 |
| PE anti-mouse CD25 antibody | Biolegend | AB_312847 |
| APC anti-mouse CD25 antibody | Biolegend | AB_2280288 |
| CD44 Monoclonal Antibody (IM7), PE | Thermo Fisher Scientific | AB_465665 |
| PE/Cyanine7 anti-mouse CD45 | Biolegend | AB_312979 |
| APC anti-mouse CD45 antibody | Biolegend | AB_312977 |
| CD45 Monoclonal Antibody, eFluor 450 | Thermo Fisher Scientific | AB_1518806 |
| Brilliant Violet 510 anti-mouse CD45 antibody | Biolegend | AB_2563061 |
| APC anti-mouse CD62L antibody | Biolegend | AB_313099 |
| Brilliant Violet 510 anti-mouse CD69 antibody | Biolegend | AB_2562326 |
| CD40 Monoclonal Antibody, PE | Thermo Fisher Scientific | AB_465649 |
| CD80 Monoclonal Antibody, PE-Cyanine7 | Thermo Fisher Scientific | AB_2573370 |
| PerCP/Cyanine5.5 anti-mouse CD86 antibody | Biolegend | AB_2074994 |
| FOXP3 Monoclonal Antibody, eFluor 450 | Thermo Fisher Scientific | AB_1518812 |
| F4/80 Monoclonal Antibody, APC | Thermo Fisher Scientific | AB_2784647 |
| Brilliant Violet 510 anti-mouse F4/80 antibody | Biolegend | AB_2562622 |
| APC anti-mouse Ki-67 antibody | Biolegend | AB_2561930 |
| PerCP/Cyanine5.5 anti-mouse Ly-6C antibody | Biolegend | AB_1659241 |
| Ly-6G Monoclonal Antibody, APC | Thermo Fisher Scientific | AB_2573307 |
| PE anti-mouse I-A/I-E antibody | Biolegend | AB_313322 |
| PE/Cyanine7 anti-mouse I-A/I-E antibody | Biolegend | AB_2069376 |
| APC anti-mouse I-A/I-E antibody | Biolegend | AB_313329 |
| APC anti-mouse IL-10 antibody | Biolegend | AB_315364 |
| NK1.1 Monoclonal Antibody, PerCP-Cyanine5.5 | Thermo Fisher Scientific | AB_914361 |
| NK1.1 Monoclonal Antibody, PE-Cyanine7 | Thermo Fisher Scientific | AB_469664 |
| PerCP/Cyanine5.5 anti-mouse Ly-6C antibody | Biolegend | AB_1659241 |
| Rat IgG2aκ Isotype Control Antibody | Thermo Fisher Scientific | AB_470051 |
| PE Rat IgG2bκ Isotype Control Antibody | Biolegend | AB_326552 |

**NMR and HRMS data of synthesized compounds**

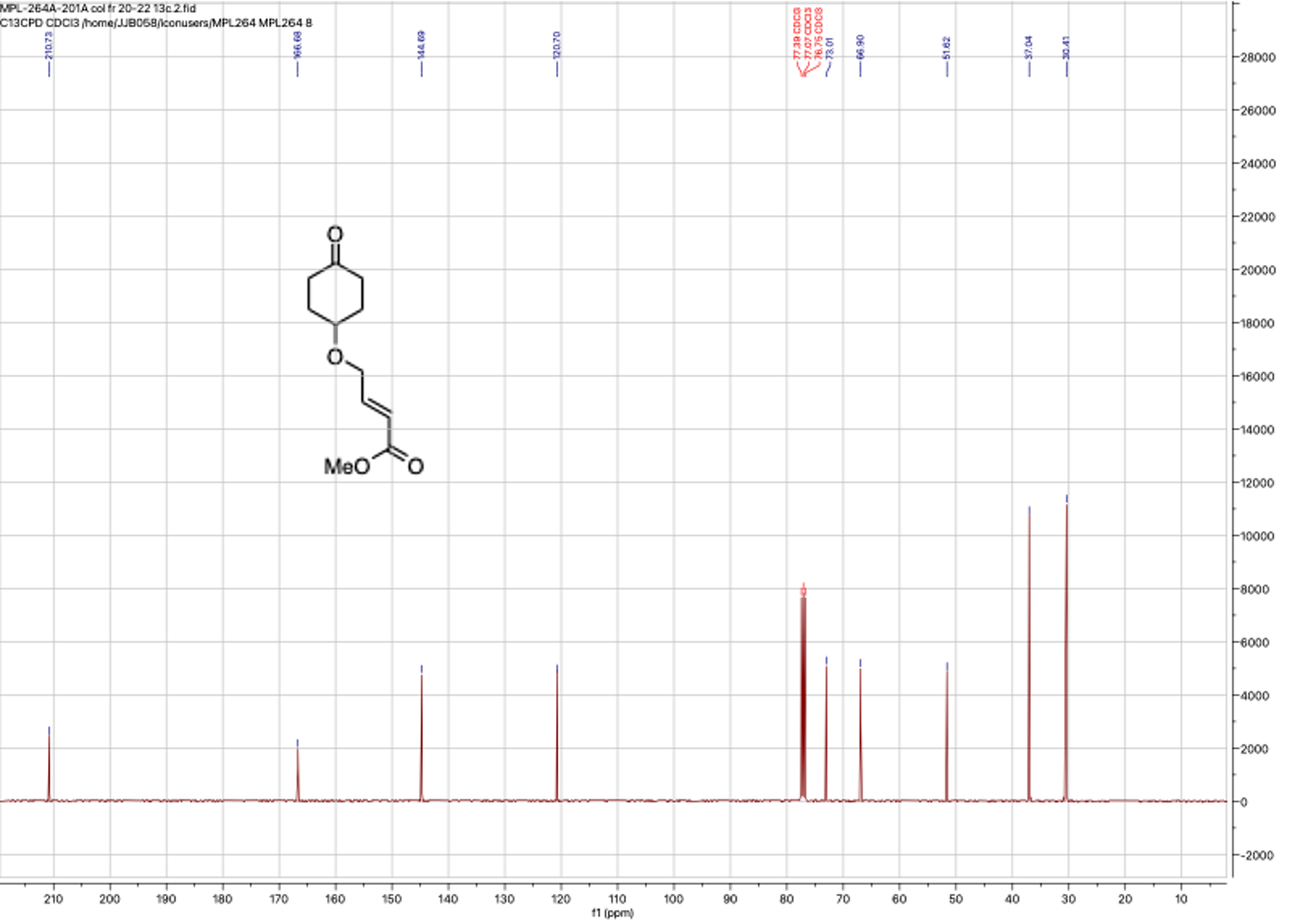


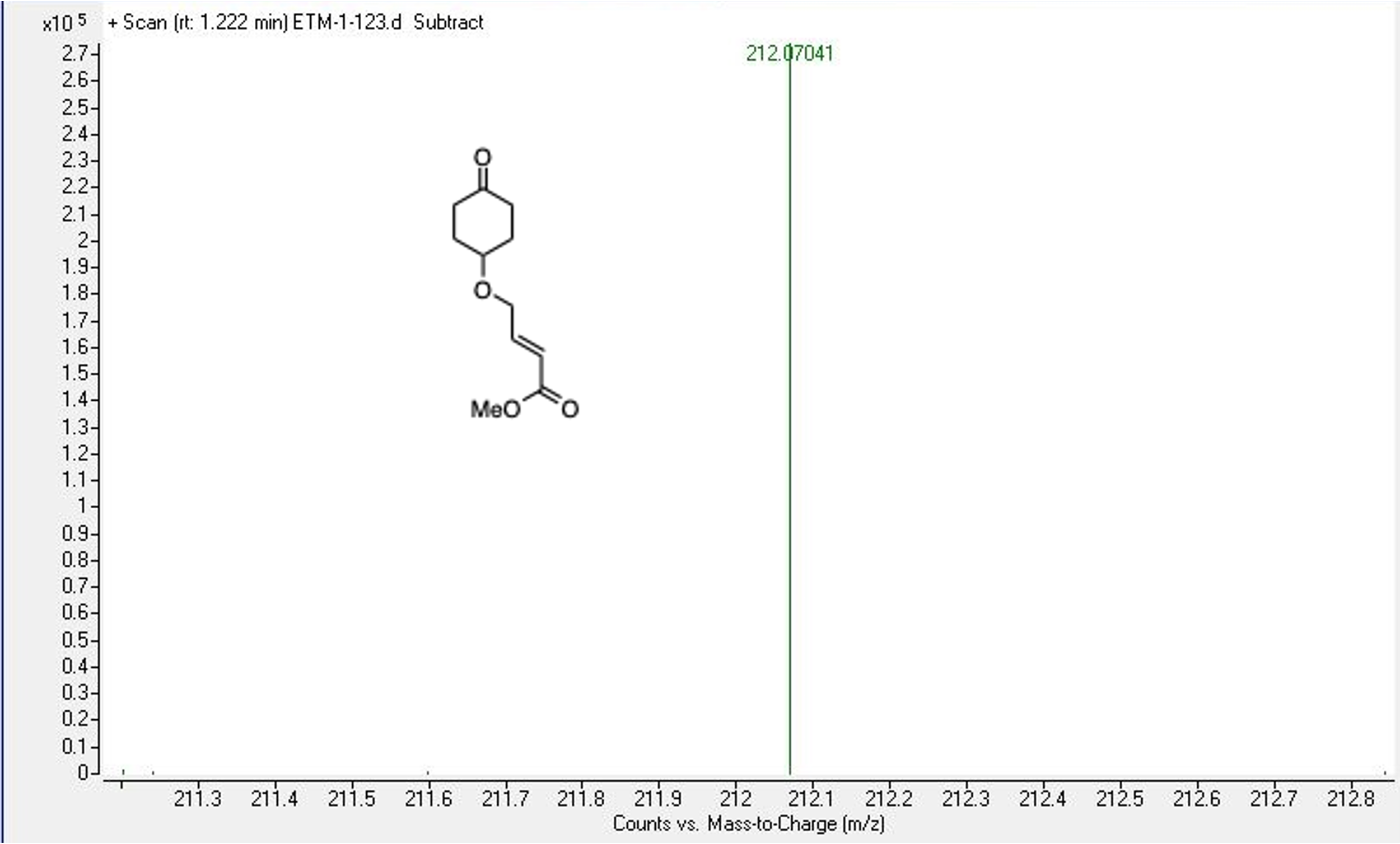

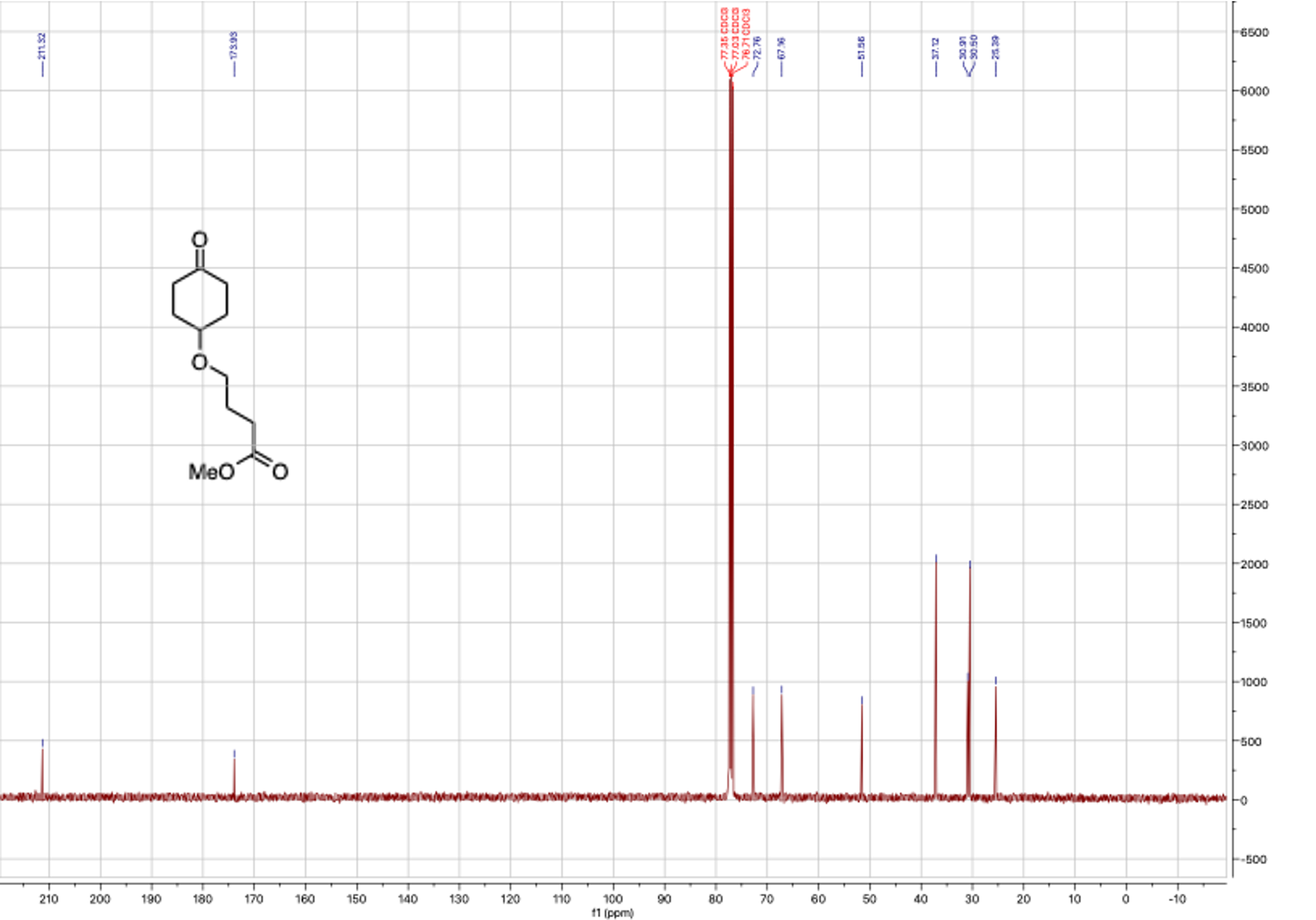

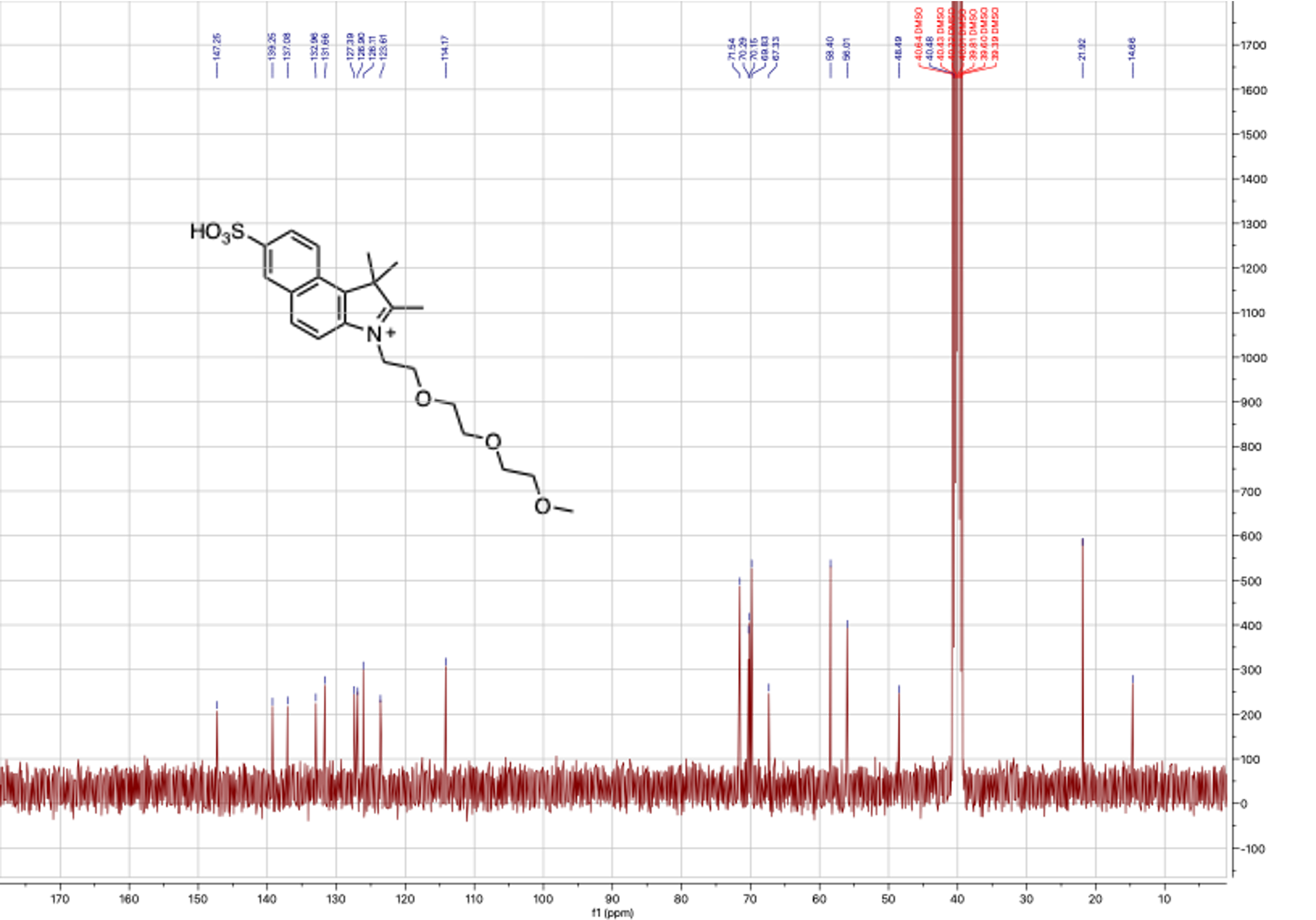


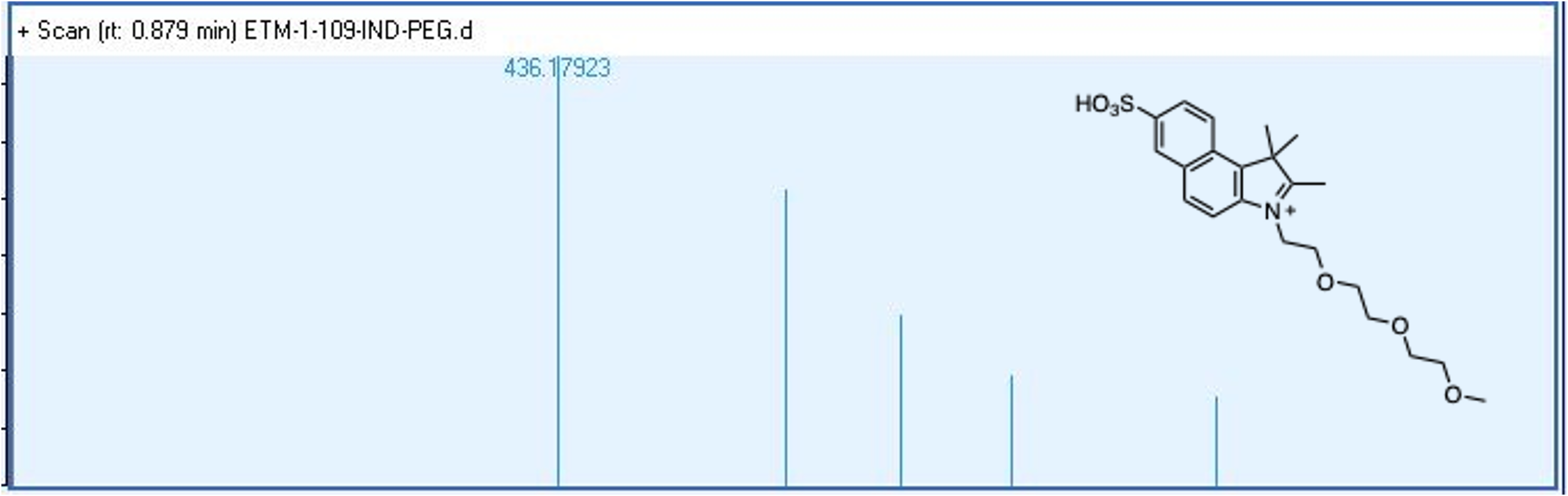


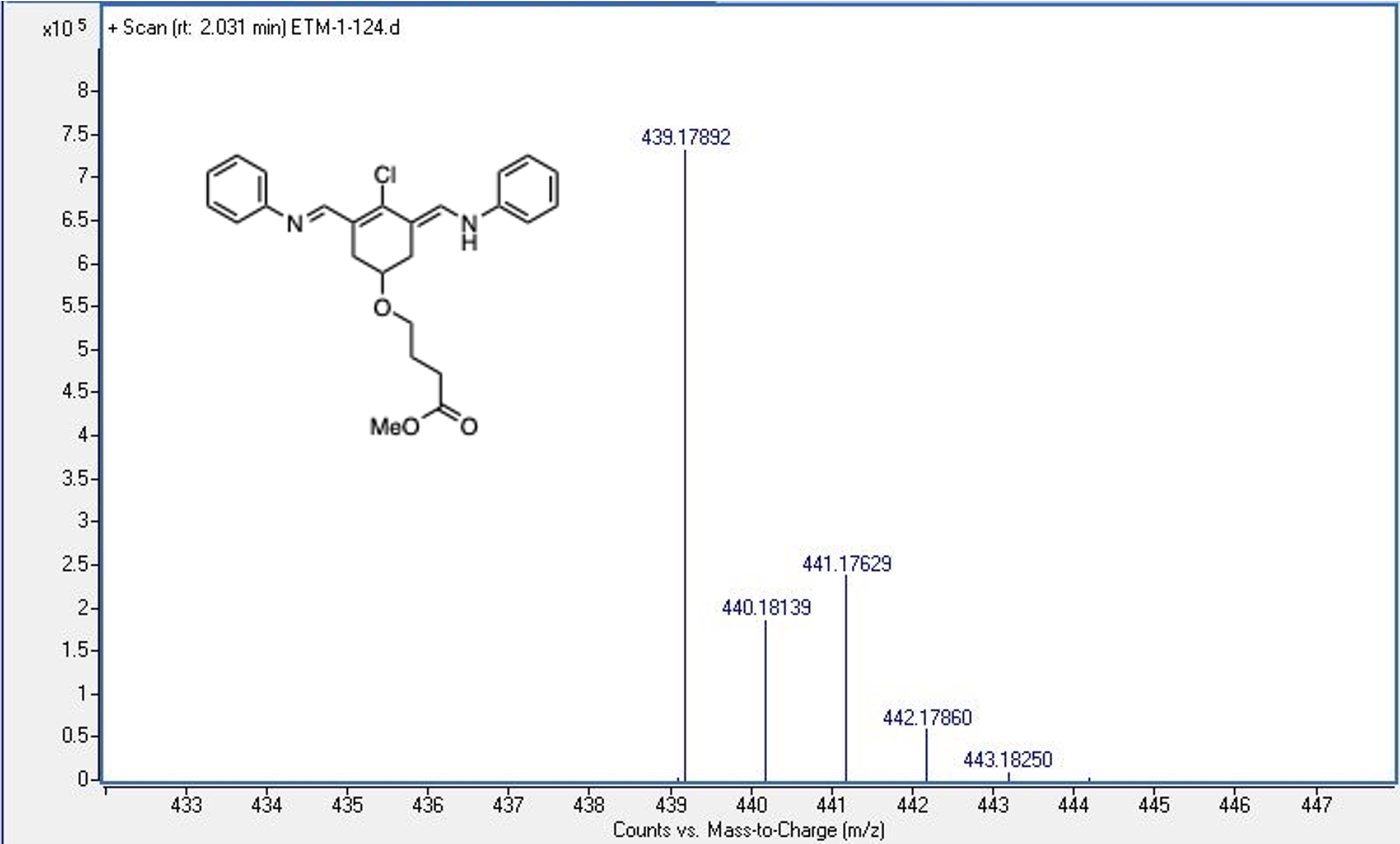

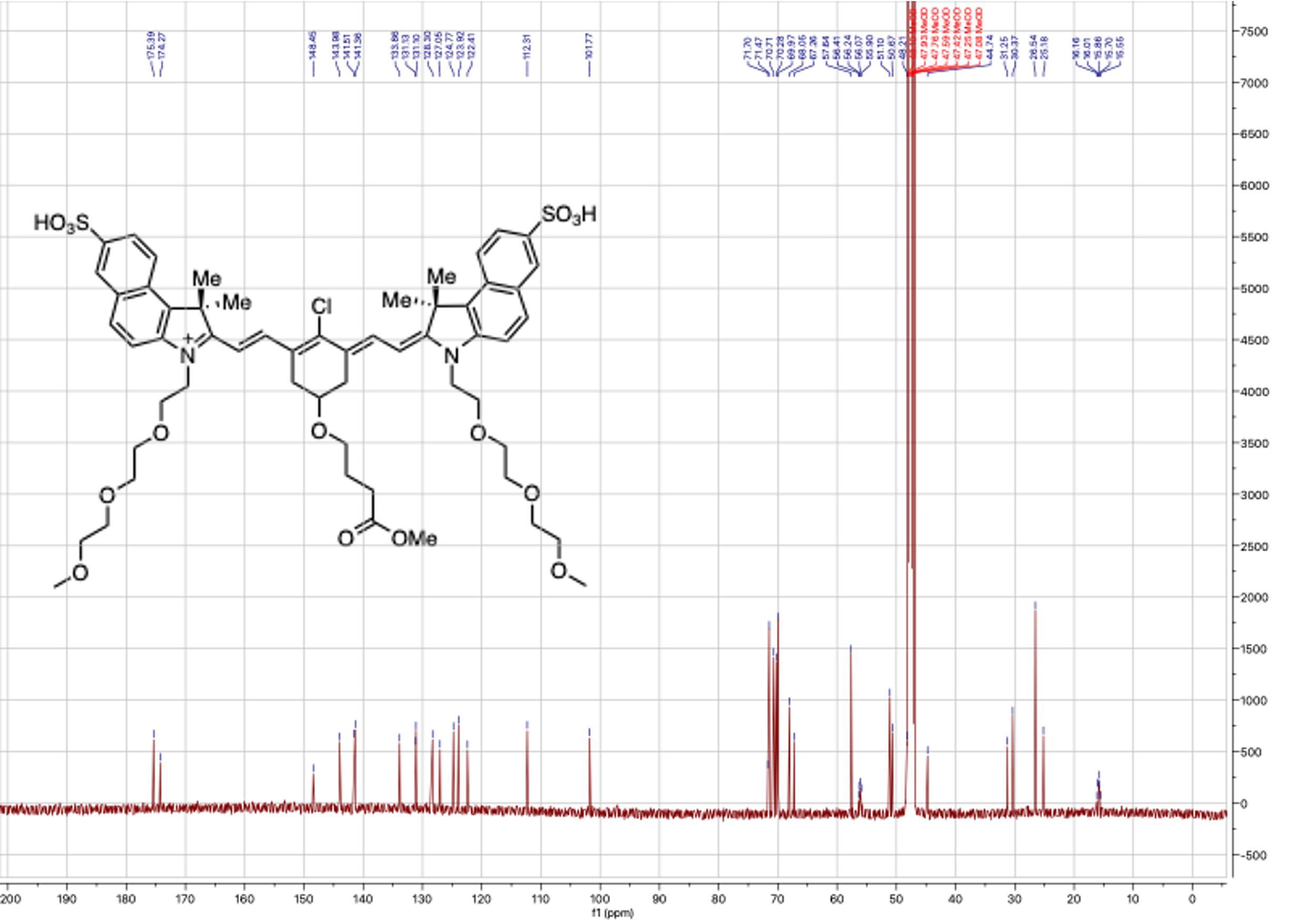


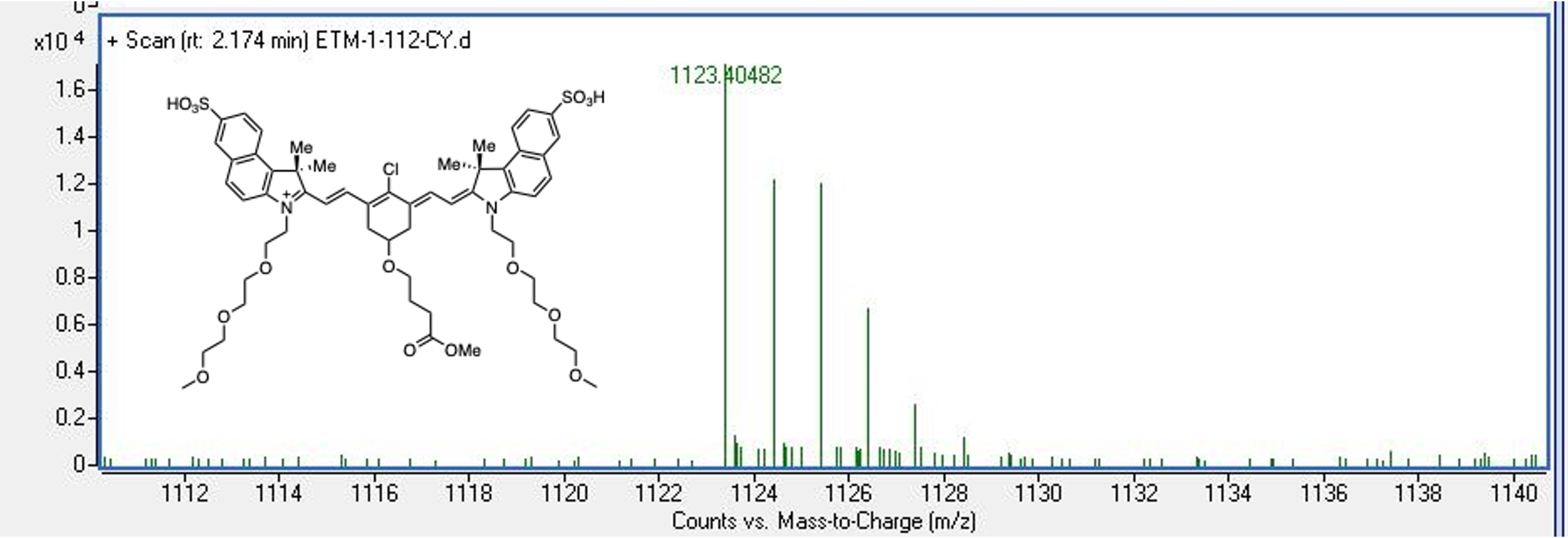

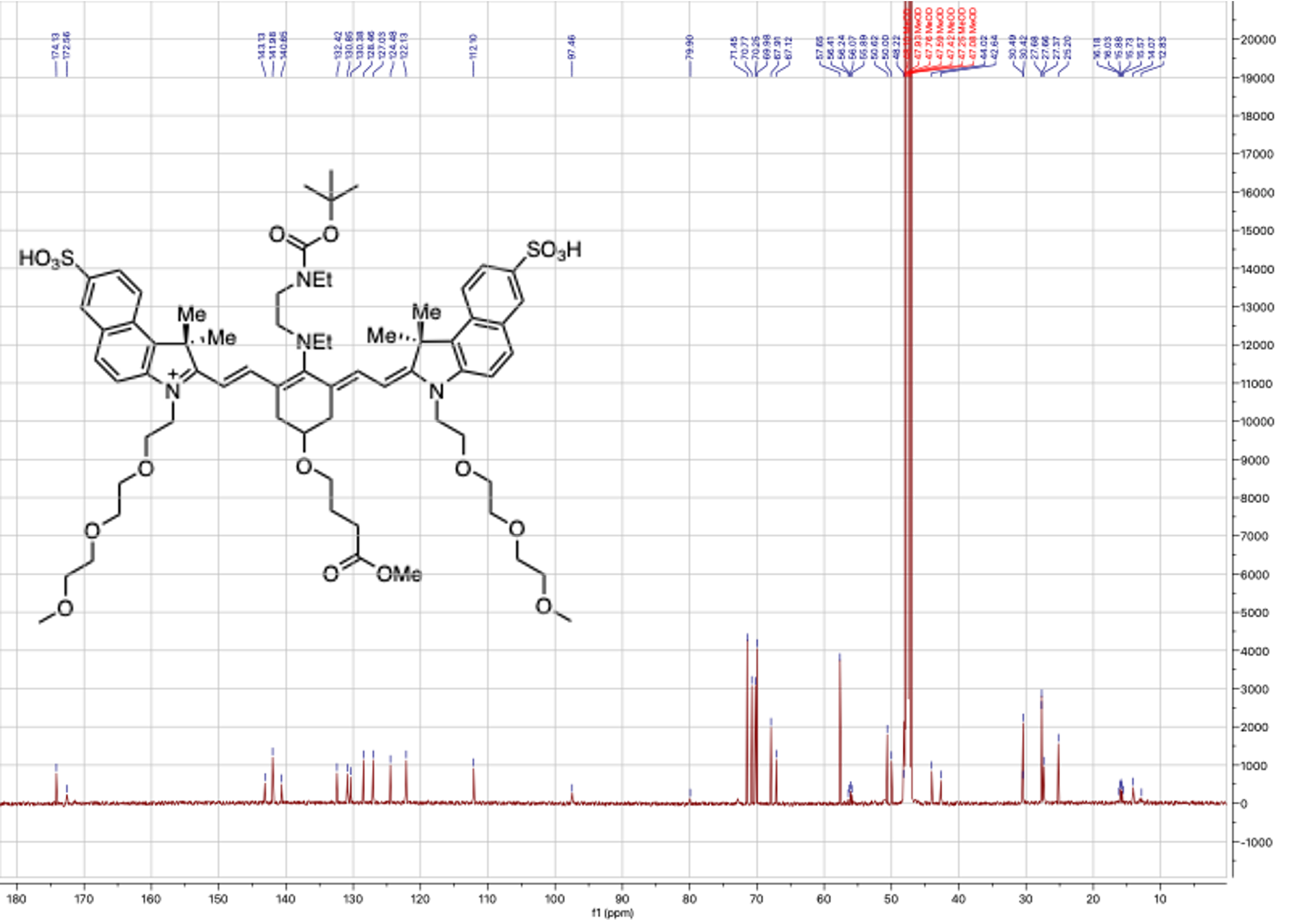

**References**

1. Malapelle A, Coslovi A, Doisneau G, Beau, J-M. An Expeditious Synthesis of N-Acetylneuraminic Acid α-C-Glycosyl Derivatives (“α-C-Glycosides”) from the Anomeric Acetates. *Eur J Org Chem* 2007;2007:3145-3157. doi: 10.1002/ejoc.200700181.
2. Mujumdar SR, Mujumdar RB, Grant CM, Waggoner AS. Cyanine-labeling reagents: sulfobenzindocyanine succinimidyl esters. *Bioconjug Chem* 1996;7:356-62. doi: 10.1021/bc960021b.
